# *Verticillium longisporum* Elicits Media-Dependent Secretome Responses With Capacity to Distinguish Between Plant-Related Environments

**DOI:** 10.3389/fmicb.2020.01876

**Published:** 2020-08-06

**Authors:** Miriam Leonard, Anika Kühn, Rebekka Harting, Isabel Maurus, Alexandra Nagel, Jessica Starke, Harald Kusch, Oliver Valerius, Kirstin Feussner, Ivo Feussner, Alexander Kaever, Manuel Landesfeind, Burkhard Morgenstern, Dörte Becher, Michael Hecker, Susanna A. Braus-Stromeyer, James W. Kronstad, Gerhard H. Braus

**Affiliations:** ^1^Department of Molecular Microbiology and Genetics, Göttingen Center for Molecular Biosciences, Institute of Microbiology and Genetics, University of Göttingen, Göttingen, Germany; ^2^Department for Plant Biochemistry, Göttingen Center for Molecular Biosciences, Albrecht-von-Haller-Institute for Plant Sciences, University of Göttingen, Göttingen, Germany; ^3^Department of Bioinformatics, Göttingen Center for Molecular Biosciences, Institute for Microbiology and Genetics, University of Göttingen, Göttingen, Germany; ^4^Department Microbial Proteomics, Institute for Microbiology, University of Greifswald, Greifswald, Germany; ^5^Department of Microbial Physiology, Institute for Microbiology, University of Greifswald, Greifswald, Germany; ^6^Michael Smith Laboratories, Department of Microbiology and Immunology, The University of British Columbia, Vancouver, BC, Canada

**Keywords:** *Verticillium longisporum*, *Verticillium dahliae*, plant- and media-dependent exoproteomes, Nep1-like proteins, effectors, plant pathogen, xylem

## Abstract

Verticillia cause a vascular wilt disease affecting a broad range of economically valuable crops. The fungus enters its host plants through the roots and colonizes the vascular system. It requires extracellular proteins for a successful plant colonization. The exoproteomes of the allodiploid *Verticillium longisporum* upon cultivation in different media or xylem sap extracted from its host plant *Brassica napus* were compared. Secreted fungal proteins were identified by label free liquid chromatography-tandem mass spectrometry screening. *V. longisporum* induced two main secretion patterns. One response pattern was elicited in various non-plant related environments. The second pattern includes the exoprotein responses to the plant-related media, pectin-rich simulated xylem medium and pure xylem sap, which exhibited similar but additional distinct features. These exoproteomes include a shared core set of 221 secreted and similarly enriched fungal proteins. The pectin-rich medium significantly induced the secretion of 143 proteins including a number of pectin degrading enzymes, whereas xylem sap triggered a smaller but unique fungal exoproteome pattern with 32 enriched proteins. The latter pattern included proteins with domains of known pathogenicity factors, metallopeptidases and carbohydrate-active enzymes. The most abundant proteins of these different groups are the necrosis and ethylene inducing-like proteins Nlp2 and Nlp3, the cerato-platanin proteins Cp1 and Cp2, the metallopeptidases Mep1 and Mep2 and the carbohydrate-active enzymes Gla1, Amy1 and Cbd1. Their pathogenicity contribution was analyzed in the haploid parental strain *V. dahliae*. Deletion of the majority of the corresponding genes caused no phenotypic changes during *ex planta* growth or invasion and colonization of tomato plants. However, we discovered that the *MEP1*, *NLP2*, and *NLP3* deletion strains were compromised in plant infections. Overall, our exoproteome approach revealed that the fungus induces specific secretion responses in different environments. The fungus has a general response to non-plant related media whereas it is able to fine-tune its exoproteome in the presence of plant material. Importantly, the xylem sap-specific exoproteome pinpointed Nlp2 and Nlp3 as single effectors required for successful *V. dahliae* colonization.

## Introduction

Vascular wilts caused by *Verticillium* spp. are widespread and destructive plant diseases, resulting in enormous economic losses. Haploid *Verticillium dahliae*, the economically most important representative of the genus, infects over 200 plant species worldwide (Klosterman et al., 2009; Inderbitzin and Subbarao, 2014). In contrast, the allodiploid *Verticillium longisporum* has a narrow host range that comprises primarily *Brassicaceae*. During the last several decades, increasing cultivation of the oilseed rape *Brassica napus* revealed *V. longisporum* as one of the most devastating pathogens of oilseed rape (Dunker et al., 2008).

*Verticillium* spp. enter the plant through the root, where the fungus then grows both inter- and intracellular in the root cortex toward the central cylinder and finally colonizes the xylem vessels (Fradin and Thomma, 2006; Eynck et al., 2007). The transpiration stream plays an essential role in supplying water and mineral salts to the aerial tissue of plants (de Boer and Volkov, 2003). The xylem sap is a nutrient-poor environment with plant defense proteins, hormones and low concentrations of amino acids and sugars (Singh et al., 2010; Carella et al., 2016). This makes it a very unique environment, which *Verticillium* spp. exploit for growth and systematic distribution in the host plant (Floerl et al., 2008; Singh et al., 2010). Factors that contribute to adaptation to the unbalanced amino acid supply include the chorismate synthase encoding gene *VlARO2* and the cross-pathway transcription factor Cpc1 (Singh et al., 2010; Timpner et al., 2013). The pathogen requires distinct sets of enzymes during different steps in plant colonization including carbohydrate-active enzymes (CAZymes) and peptidases, as well as small secreted proteins, to establish an infection and overcome the immune response of the plant. Several extracellular proteins including polygalacturonases, pectate lyases, xylanases or lipases presumably contribute to virulence during pathogen−host interactions (Isshiki et al., 2001; Yakoby et al., 2001; Oeser et al., 2002; Voigt et al., 2005; Brito et al., 2006).

The plant immune response depends in part on transmembrane receptor proteins termed pattern recognition receptors. These cell surface localized receptors recognize conserved microbial molecules and structural motifs designated as pathogen-associated molecular patterns; examples include the fungal cell-wall polymer chitin (Sorrell and Chen, 2009). Perception of such patterns elicits a basal defense response which halts colonization by non-adapted pathogens and results in pathogen-associated molecular patterns-triggered immunity. Host-adapted pathogens circumvent these plant defense responses by secretion of specific effector proteins as virulence factors for different phases of the infection cycle (Dodds and Rathjen, 2010). These secreted effectors may act passively or actively to combat plant defense responses (Lo Presti et al., 2015).

Well known examples of fungal effectors include the Avr4 and Ecp6 effectors from the leaf mold fungus *Cladosporum fulvum* that bind to chitin oligosaccharides via a carbohydrate-binding module or LysM domain, respectively (van Esse et al., 2007; de Jonge et al., 2010; Lo Presti et al., 2015; Volk et al., 2019). Similarly, a chitin scavenging function has also been assigned to Cp1 in *V. dahliae* strain XH-8. *CP1* knockout mutants were affected in cotton virulence (Zhang et al., 2017). This chitin protection leads to the suppression of the pathogen-associated molecular patterns-triggered immunity of the plant and shields the fungal cell wall from plant chitinases that hydrolyze chitin (van Esse et al., 2007; de Jonge et al., 2010; Lo Presti et al., 2015; Volk et al., 2019). Other fungal effectors such as metalloproteases possess enzymatic activity and are able to truncate plant chitinases that attack the fungal cell wall (Naumann et al., 2011; Han et al., 2019). Toxins provide another means for pathogens to attack plant hosts. For example, Nep1-like proteins (NLPs) with the necrosis-inducing *Phytophtora* protein 1 (NPP1) domain induce immune responses and cell death in host tissues and are conserved among fungi including *Verticillium* spp. (Gijzen and Nürnberger, 2006; Santhanam et al., 2013). *V. dahliae* isolates encode up to eight NLP homologs (Zhou et al., 2012; Santhanam et al., 2013) whereas most other fungi generally only possess up to three NLPs (Gijzen and Nürnberger, 2006). Only Nlp1 and Nlp2 show cytotoxic activity in *Nicotiana benthamiana* leaves and play strain- and host-specific roles in *V. dahliae* virulence (Zhou et al., 2012; Santhanam et al., 2013). Nlp1 and Nlp2 are required for *V. dahliae* JR2 pathogenicity on tomato and *A. thaliana* (Santhanam et al., 2013) whereas the corresponding proteins in *V. dahliae* V592 did not alter virulence on cotton (Zhou et al., 2012). Plant pathogens additionally require a set of CAZymes that facilitate the breakdown of the plant cell wall (Marton et al., 2018). The genomes of *Verticillium* species encode a greater number of CAZymes with a strikingly high repertoire of pectin-degrading enzymes compared to the secreted proteins of other plant pathogens (Klosterman et al., 2011; Lo Presti et al., 2015).

As the fungus lives in the vascular tissues during most of its life cycle in the plant, further knowledge about specific secretion responses would enable a better understanding of fungal−plant interactions during the infection process. Once the fungus resides inside the plant, it is inaccessible for pesticides and therefore, the management of Verticillium wilt is very challenging. The most effective and widely used soil fumigants, methyl bromide or metam sodium, are used for high valuable crops, but are not profitable for all crops. Furthermore, these and other banned fungicides, are associated with environmental issues (Klosterman et al., 2009; Yellareddygari and Gudmestad, 2018). Therefore, an indispensable approach for protection is to use resistant plant varieties, but these are not available for most crops. The selection pressure on fungal strains to quickly overcome genetic resistances of the plant makes it even more difficult to develop new resistant varieties (Klosterman et al., 2009; Lo Presti et al., 2015). Consequently, an increased understanding of the infection process for *Verticillium* spp. is necessary to identify new approaches for disease control.

Until now, it is not known how the effector repertoire of *Verticillium* spp. is expressed once the pathogen enters the plant. Analysis of *Verticillium* strains in their vascular environment is technically demanding, and a large number of plants are required to harvest sufficient amounts of xylem sap. Proteomic approaches can be fruitful because comparison of the intracellular fungal proteome in diluted xylem sap and pectin-rich medium resulted in the identification of a disease-related catalase peroxidase, which was only up-regulated in the presence of xylem sap and not in the presence of pectin (Singh et al., 2012).

In this study, we extended the comparative analysis with rapeseed xylem sap and focused on the fungal secretome. *V. longisporum* secreted proteins that were derived from cultivation in different growth media were identified by a proteomic approach and the protein patterns induced by different environments were compared. Our goal was to obtain a more comprehensive overview of the secreted factors of *V. longisporum* in response to different substances in its environment that putatively reflect different stages of the infection. We analyzed the exoproteomes of *V. longisporum* on a broad range of media from water to minimal and complete media. As an additional condition, we applied simulated xylem medium (SXM), which is rich in pectin and which was originally developed to mimic the natural plant growth environment, the xylem sap (Neumann and Dobinson, 2003). All exoproteomes were compared to fungal cultures grown in extracted xylem sap of the *V. longisporum* host oilseed rape *B. napus*.

Our results demonstrate that *V. longisporum* is able to distinguish between the different environments to express different secretome patterns. The pectin-rich medium and xylem sap each triggered distinct protein patterns in comparison to all other tested media. The fungal response to growth in the pectin-rich medium and xylem sap consists of a shared core exoproteome and an additional group of uniquely secreted proteins. A small number of proteins are specifically expressed in xylem sap including CAZymes and other potential virulence factors. Of the factors that were specifically enriched in xylem sap, the NLPs Nlp2 and Nlp3 we demonstrate contributions to plant pathogenicity as virulence factors.

## Results

### Xylem Sap and Pectin-Rich SXM Trigger Specific Exoproteomic Patterns Compared to Other Growth Media

**FIGURE 1:**
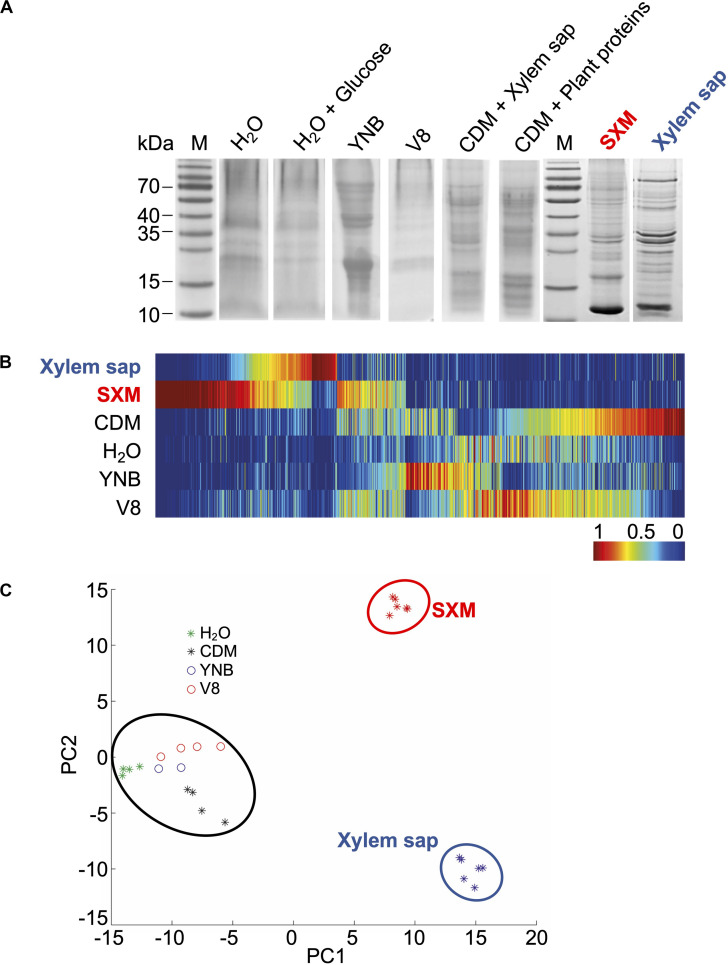
Exoproteome signatures of *V. longisporum* in different growth media. *V. longisporum* 43 was cultivated in complete medium (PDM) for 4 days before the sedimented mycelia and spores were dissolved in different media (dH_2_O, dH_2_O + 0.1% glucose, yeast nitrogen base (YNB), 10% vegetable juice (V8), minimal medium (CDM) supplemented with 7% xylem sap or plant proteins, simulated xylem medium (SXM) and extracted xylem sap from *B. napus*) and cultivated for four more days. **(A)** Precipitated proteins from the supernatants were separated by one-dimensional SDS–PAGE. The colloidal Coomassie stained exoproteome samples on gel displayed a strong variation between the protein band distributions of all different culture conditions. Lanes M represent the molecular weight marker. Complete lanes of the *V. longisporum* exoproteomes were subjected to tryptical protein digestion and the resulting peptides were analyzed by LC-MS/MS. **(B)** Clustering analysis of protein abundances (spectral counts) was facilitated by MarVis-Suite tools and is visualized as one-dimensional self-organizing maps. Rows represent the compared growth conditions. The spectral counts were normalized and color-coded according to the indicated scale. Red indicates increased, dark blue no spectral counts. **(C)** The principle component analysis plot of the exoproteomes based on the spectral counts was performed with MarVis-Suite software (Meinicke et al., 2008). Each dot represents one independent culture. The compared exoproteome signatures cluster in three groups: a first cluster is formed by all xylem sap culture samples (blue circle); the second cluster contains all SXM culture samples (red circle) and the third cluster consists of all other samples (black circle).

*Verticillium longisporum* is a rapeseed pathogenic fungus that is able to grow on a variety of different substrates and colonizes the xylem vessels of plants. This requires the adaptation of the fungus to changing nutrient conditions and other biotic and abiotic factors. We examined how different growth media affected the exoproteome of *V*. *longisporum* with a specific focus on identification of distinct patterns triggered by different plant-related contents. For all experiments, *V. longisporum* was precultured in liquid potato dextrose medium to ensure an equal initial growth state prior to the media-specific induction of secretion. The proteins of the *V. longisporum* culture supernatants from different media were precipitated and separated by one-dimensional SDS−PAGE thereby resulting in several different patterns in colloidal Coomassie stained gels (Figure 1A). The defined media conditions corresponded to different levels of complexity including nutrient-free water, water with glucose as carbon source, and a more complex nitrogen-rich medium (YNB: Yeast Nitrogen Base). Several plant-related media were included because the natural habitat of the pathogen *V. longisporum* is inside host plants. These media included the nutrient-limited sucrose medium CDM (Czapek-Dox medium), which was either supplemented with 7% of *B. napus* xylem sap or plant proteins, 10% vegetable juice (V8 juice) and the pectin-rich SXM, which was developed to mimic fungal growth conditions in plants *in vitro* (Neumann and Dobinson, 2003; Mandelc and Javornik, 2015). Finally, we also used extracted xylem sap from the rapeseed plant *B. napus*, which was filtered, excluding proteins larger than 10 kDa but including smaller plant proteins and signal molecules.

To obtain a more comprehensive analysis, complete lanes of the gels were fractionally subjected to tryptic protein digestion and the resulting peptides were analyzed by liquid chromatography-tandem mass spectrometry (LC-MS/MS). The obtained raw data were channeled through a bioinformatics pipeline based on Proteome Discoverer Software 1.3^TM^ and an in-house genome-wide protein sequence database of *V. longisporum*. The received spectral counts were compared on single secreted protein level by color-coded one-dimensional self-organizing maps (Figure 1B). These revealed that proteins that were strongly enriched in xylem sap or SXM were not enriched in any other condition. Differences in the exoproteome signatures are also illustrated by sample clustering in a principle component analysis plot (Figure 1C). Exoproteomes of *V. longisporum* derived from very diverse media including nutrient-free water, V8 juice, CDM or YNB medium show a similar pattern. Supplementation of CDM with *B. napus* plant proteins or xylem sap with a final concentration of 7% did not result in a different exoproteome pattern, neither did glucose supplementation to water. Therefore, the respective results for these conditions were combined together. In contrast, proteins secreted in pectin-rich SXM or xylem sap each showed a distinct pattern in comparison to the other media conditions. These patterns representing the latter exoproteomes are similar in some features as the clusters lie close to each other on the x-axis, although some differences are present as analyzed further below.

Overall, our analysis illustrates that the fungus has the potential to form a general secretome response to non-plant related environments and, in addition, a similar, but more specialized response to plant-related substances (Figure 1C).

### Pectin-Rich Medium and Xylem Sap Elicit Distinct *V. longisporum* Exoproteome Responses

Similarities and differences of the specific *V*. *longisporum* exoproteome responses in the pectin-rich SXM compared to xylem sap were analyzed in more detail. The data set was filtered with a statistical workflow using MarVis-Suite (Kaever et al., 2012). For our approach we focused on the secretome without extracellular vesicles to test whether conventionally secreted proteins are xylem sap specific. Therefore, we filtered for predicted extracellular localization and the possession of a signal peptide. Supplementary Table S1 shows the identities of the 441 proteins from an in-house database with their protein sequences and identified peptides. The list is sorted according to the most specifically enriched proteins in xylem sap (green) and SXM (red), respectively. Proteins that are not considered as specifically enriched are listed at the bottom of the table and belong to the core exoproteome. Putative unconventionally secreted proteins that were detected in xylem sap and SXM are listed in Supplementary Tables S2, S3, respectively.

**FIGURE 2:**
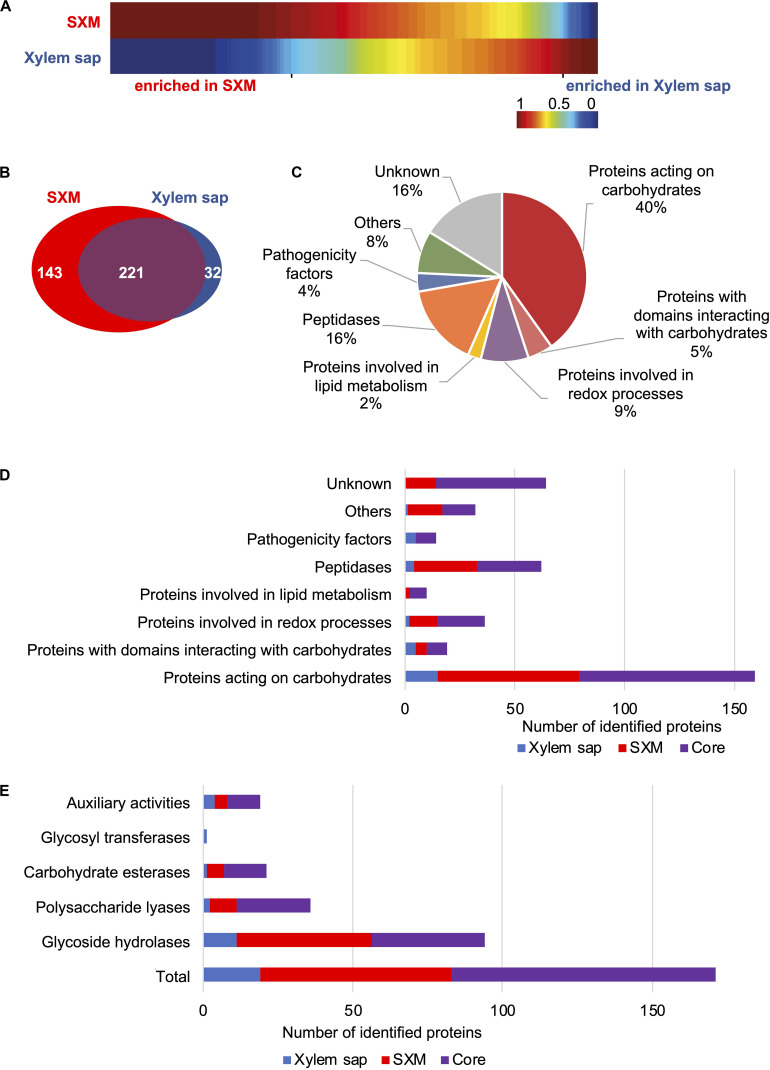
Comparison of exoproteome signatures for *V. longisporum* growth in xylem sap and pectin-rich simulated xylem medium. The exoproteome of *V. longisporum* upon cultivation in simulated xylem medium (SXM) and xylem sap was compared. A list of identified proteins is found in Supplementary Table S1. **(A)** Clustering analysis of protein abundances (spectral counts) is visualized as one-dimensional self-organizing maps, which was facilitated by the software MarVis-Suite. Upper and lower rows represent the two compared growth conditions. Each column corresponds to spectral counts of one identified protein. The spectral counts were normalized and color-coded according to the indicated scale. Red indicates increased, dark blue no spectral counts. **(B–E)** BLAST searches of identified *V. longisporum* proteins in panel **(A)** against the *V. dahliae* JR2 and the *V. alfalfae* VaMs.102 proteomes from Ensembl Fungi were conducted and functional analysis is based on their protein sequences. **(B)** The Venn diagram displays the number of proteins specifically enriched in xylem sap (blue) and proteins enriched in SXM (red). Similarly enriched proteins in both media form the core exoproteome (violet). **(C)** The cake diagram shows the functional classification of the 396 identified secreted proteins into main protein groups according to their predicted domains. **(D)** The functional groups are presented with the number of identified proteins in the different cultivation environments. **(E)** Classification by CAZyme modules is shown for each cultivation type.

Clustering analysis of spectral count data of the 441 proteins with MarVis-Suite was visualized as a one-dimensional self-organizing map (Figure 2A). A set of proteins, which have a stable abundance in both conditions is considered as the shared core exoproteome. Proteins that showed different peptide counts in the two growth conditions were considered as differentially enriched (Figure 2A, “enriched in Xylem sap” and “enriched in SXM,” respectively).

*Verticillium longisporum* is an allodiploid organism derived from two parental strains, and *V. longisporum* 43 used in this study is a result of an A1xD1 hybridization event. A1 and D1 are described as so far unknown haploid *Verticillium* species, of which D1 is closely related to *V. dahliae* and A1 is distantly related to *V. alfalfae* (Inderbitzin and Subbarao, 2014). Most genes are encoded in two copies, reflecting the two isogenes of both parental lineages. BLAST searches against the *V. dahliae* JR2 and the *V. alfalfae* VaMs.102 proteomes from Ensembl Fungi were conducted. As a consequence, two isogene products were detected for most identified proteins.

The list of proteins was further and thoroughly analyzed manually to functionally classify the candidates. As the Ensembl Fungi annotations are more robust, further analyses are based on the *V. dahliae* JR2 protein sequences except for candidates with no corresponding hit in *V. dahliae*. Here, the protein sequences for further analyses were retrieved from the *V. alfalfae* VaMs.102 proteome from Ensembl Fungi. Putative functions of robust annotated proteins were addressed with InterProScan, the CAZy database and dbCAN2. All details are given in Supplementary Table S4. The Venn diagram in Figure 2B displays the 396 candidates with robust annotations in different groups. Protein extracts from pectin-rich SXM and xylem sap share a core exoproteome of 221 proteins with a similar abundance in both media, but each also induced the secretion of distinct exoprotein patterns. SXM cultivation resulted with 143 secreted proteins in a four-fold higher number of secreted proteins specifically enriched in comparison to xylem sap, where the peptide count of 32 proteins was specifically increased (Figure 2B).

These results show that SXM, which is used to simulate xylem sap *in vitro*, and xylem sap of *B. napus* induce distinct secretion responses with different facets. This indicates that the fungus is able to fine-tune its secretion responses.

### *Verticillium longisporum* Secretes a Broad Arsenal of Substrate-Degrading Enzymes in Pectin-Rich Medium and in Xylem Sap

All 396 identified secreted proteins were classified into functional groups according to the predicted domains (Figure 2C and Supplementary Table S4). For 65 identified proteins, classified as hypothetical gene products, no information about structural domains or putative functions could be found (“Unknown”). “Proteins involved in lipid metabolism,” “Pathogenicity factors,” and smaller groups combined as “Others” represent minor groups, whereby “Pathogenicity factors” represent a group of proteins containing domains of well-known effectors. More proteins were sorted to “Proteins involved in redox processes” (9%) or “Peptidases”(16%) whereas the functional classification revealed an overrepresentation of proteins involved in carbohydrate metabolism or catabolism with around 40% of proteins acting on carbohydrates and another 5% of proteins with domains interacting with carbohydrates (Figure 2C and Supplementary Table S5).

Further analysis of proteins from functional groups regarding the induction by different media showed that pectin-rich medium predominantly triggered the secretion of carbohydrate-degrading enzymes, but also peptidases and redox enzymes (Figure 2D and Supplementary Table S5). In the SXM-specific exoproteome no proteins were grouped as “Pathogenicity factors.” Cultivation in xylem sap triggered the unique secretion of carbohydrate-degrading enzymes, pathogenicity factors, peptidases and redox enzymes though the total number of enriched proteins is significantly smaller compared to the SXM-specific secreted proteins.

The majority of identified secreted proteins comprise the CAZymes, which contain protein motifs that have been classified into sequence-related families of CAZyme modules (Lombard et al., 2014). Within the group of all secreted proteins we identified 94 glycoside hydrolases (GHs), 36 polysaccharide lyases (PLs), 21 carbohydrate esterases (CEs), one glycosyltransferase (GT), 19 auxiliary activities (AAs). Of these CAZymes, 19 proteins additionally possess non-catalytic carbohydrate-binding modules (Figure 2E and Supplementary Table S6). In the proteins from the pectin-rich medium condition, the CAZymes are highly represented with 64 proteins whereas in xylem sap only 19 CAZymes are specifically enriched and 88 proteins belong to the core exoproteome (Supplementary Table S6). The core exoproteome exhibits an overrepresentation of CAZyme families with 32 proteins acting on pectin, including members of family GH28 (five proteins), PL1 (16 proteins), PL3 (seven proteins) and CE8 (four proteins). Additionally, the SXM-specific and most enriched CAZyme families comprise 20 pectin-degrading enzymes (families GH28, PL1 and CE12 with ten, six and four proteins, respectively, Supplementary Table S6). Only a few CAZymes were specifically enriched in the xylem sap condition, and these were distributed in different families.

Overall, we found that *B. napus* xylem sap and pectin-rich SXM, employed as plant-related culture environments, predominantly induced the secretion of carbohydrate-degrading enzymes. Compared to the rapeseed xylem sap condition, SXM triggered an additional set of CAZymes that were specifically enriched after cultivation in this medium.

### Xylem Sap Triggers the Secretion of Potential and Known Verticillium Effectors

Compared to the pectin-rich SXM, *V. longisporum* formed a more specific secretion response in xylem sap with only 32 proteins that are uniquely enriched. This indicates that the fungus can distinguish between xylem sap and the presence of other plant material and accordingly fine-tunes its protein secretion. Furthermore, the proteins secreted in the host xylem sap might be specifically important during plant colonization.

Table 1 displays the xylem sap-specific proteins. The corresponding isogenes are paired up and the best hit in *V. dahliae* JR2 is given, except for the *V. alfalfae* specific proteins that were searched against the *V. alfalfae* VaMs.102 proteome (Ensembl Fungi). Within the identified groups, proteins were ranked according to the quotient of peptide counts identified in xylem sap (XyS) by the number detected in the pectin-rich SXM. Displayed peptide counts were averaged from six replicates. If the number was below 1, it was calculated as 0 and the quotient was given as the average XyS peptide counts (>). The 32 xylem sap-specific proteins comprise 15 “Proteins acting on carbohydrates,” five “Proteins with domains interacting with carbohydrates,” five “Pathogenicity factors,” four “Peptidases,” two “Proteins involved in redox processes” and one with a ubiquitin binding domain that was grouped as “Other” (Table 1).

**TABLE 1:** Xylem sap-specific exoproteins of *V. longisporum*.

	Name	Vl43 identifier	Best Hit *V. dahliae* JR2	Protein domain	Peptide counts		XyS/SXM	s/l ratio
					SXM	XyS		
Proteins acting on carbohydrates	Gla1	vl43-au16.g17458.t1	VDAG_JR2_Chr8g11020a	Glycoside hydrolase 15,	0	8.2	>8.2	0.76
	Gla1	vl43-au16.g13024.t1	VDAG_JR2_Chr8g11020a	Carbohydrate binding module family 20	0	3.8	>3.8	0.62
	Amy1	vl43-au16.g6892.t1	VDAG_JR2_Chr7g03330a	Glycoside hydrolase 13	1.5	12	8	0.68
		vl43-au16.g9360.t1	*V. alfalfae*: VDBG_05827	Glycosyl hydrolases 134	0	7.5	>7.5	0.77
		vl43-au16.g6746.t1	VDAG_JR2_Chr3g11080a	Glycoside hydrolase 43	1.2	7.8	6.5	0.68
		vl43-au16.g12522.t1	VDAG_JR2_Chr3g11080a	Glycoside hydrolase 43	1	5.8	5.8	0.61
		vl43-au16.g12596.t1	VDAG_JR2_Chr6g04040a	Glycosyl hydrolases 11	0.8	5.2	>5.2	0.55
		vl43-au16.g15027.t1	VDAG_JR2_Chr6g09340a	Polysaccharide lyase 6	2.2	11.2	5.1	0.57
		vl43-au16.g5309.t1	VDAG_JR2_Chr6g09340a	Polysaccharide lyase 6	2.3	10.8	4.7	0.59
		vl43-au16.g207.t1	VDAG_JR2_Chr1g06940a	Glycoside hydrolase 16	2.2	9	4.1	0.56
		vl43-au16.g14986.t1	VDAG_JR2_Chr1g06940a	Glycoside hydrolase 16	2.3	9.5	4.1	0.55
		vl43-au16.g15947.t1	VDAG_JR2_Chr3g00250a	Glycoside hydrolase 131	1	4	4	0.43
		vl43-au16.g9097.t1	*V. alfalfae*: VDBG_03110	Glycoside hydrolase 12	1	2.8	2.8	0.38
		vl43-au16.g16765.t1	VDAG_JR2_Chr1g04240a	Glycosyl transferase 1	0.3	1.7	>1.7	0.49
		vl43-au16.g9025.t1	VDAG_JR2_Chr7g01210a	Carbohydrate esterase 1, Carbohydrate binding module family 1	0.2	1.3	>1.3	0.49
Proteins with domains interacting with carbohydrates	Cbd1	vl43-au16.g11636.t1	VDAG_JR2_Chr4g04440a	Auxiliary activity 13, Carbohydrate binding module family 20	1.7	13.8	8.1	0.71
	Cbd1	vl43-au16.g3945.t1	VDAG_JR2_Chr4g04440a		1.3	6.8	5.2	0.55
		vl43-au16.g5519.t1	VDAG_JR2_Chr6g09790a	Auxiliary activity 9	2	8.2	4.1	0.51
		vl43-au16.g20506.t1	VDAG_JR2_Chr6g09790a	Auxiliary activity 9	2.3	8.8	3.8	0.46
		vl43-au16.g11273.t1	VDAG_JR2_Chr6g05220a	WSC Carbohydrate binding domain	3.2	11.7	3.7	0.46
Pathogenicity factors	Nlp3	vl43-au16.g9727.t1	VDAG_JR2_Chr4g05950a	Necrosis inducing protein	0.3	8.3	>8.3	0.84
	Nlp3	vl43-au16.g96.t1	VDAG_JR2_Chr4g05950a	Necrosis inducing protein	0.2	6.3	>6.3	0.81
	Nlp2	vl43-au16.g3884.t1	VDAG_JR2_Chr2g05460a	Necrosis inducing protein	1	4.8	4.8	0.61
	Nlp2	vl43-au16.g12566.t1	VDAG_JR2_Chr2g05460a	Necrosis inducing protein	1.8	6.5	3.6	0.47
	Cp2	vl43-au16.g16459.t1	VDAG_JR2_Chr2g07000a	Cerato-platanin	0.3	1.8	>1.8	0.41
Peptidases	Mep2	vl43-au16.g19470.t1	VDAG_JR2_Chr1g21900a	Peptidase M43	1	8.8	8.8	0.57
	Mep2	vl43-au16.g11262.t1	VDAG_JR2_Chr1g21900a	Peptidase M43	1.3	8.7	6.7	0.55
	Mep1	vl43-au16.g19320.t1	VDAG_JR2_Chr8g09760a	FTP domain, Peptidase M36, fungalysin	1.7	14	8.2	0.69
	Mep1	vl43-au16.g14388.t1	VDAG_JR2_Chr8g09760a		1.8	13.5	7.5	0.65
Proteins involved in redox processes		vl43-au16.g2684.t1	VDAG_JR2_Chr8g10630a	FAD-binding domain	6	13.2	2.2	0.33
		vl43-au16.g13340.t1	VDAG_JR2_Chr8g10630a	FAD-binding domain	8	15.3	1.9	0.34
Other		vl43-au16.g18734.t1	VDAG_JR2_Chr1g04640a	Ubiquitin 3 binding protein But2, C-terminal	9.7	18.8	1.9	0.37

*Vl43, V. longisporum 43; SXM, simulated xylem medium; XyS, xylem sap; s/l, signal-to-level; FTP, fungalysin/thermolysin propeptide; WSC, putative carbohydrate-binding domain.*

Several potential virulence factors were identified in the xylem sap-specific response set. Proteins involved in the degradation of carbohydrates are known to contribute to *V. dahliae* pathogenicity (Gui et al., 2017; Yang et al., 2018). The five proteins comprising domains of already characterized *V. dahliae* effectors incorporate either NPP1 or cerato-platanin (CP) domains. NPP1 domains are characteristic for necrosis and ethylene inducing protein-like proteins (NLP) of which *Verticillium* spp. contain up to eight members (Santhanam et al., 2013). Nlp1 and Nlp2 were previously shown to differentially contribute to *V. dahliae* pathogenicity on different hosts (Wang et al., 2004; Zhou et al., 2012; Santhanam et al., 2013). Our secretome approach identified four isogene products corresponding to two NLPs, Nlp2 and Nlp3. Of the CP domain-containing proteins, one isogene assigned to Cp2 was identified as specifically enriched in xylem sap. *V. dahliae* possesses two CP proteins of which Cp1 affects virulence on cotton (Zhang et al., 2017). Additionally, four isogenes of two metallopeptidases were found in the xylem sap-specific secretome. Metalloproteases are able to truncate host defense proteins such as chitinases and therefore have the potential to act as virulence factors (Naumann et al., 2011).

These findings show that *V. longisporum* finetunes its protein secretion response in the host xylem sap, which include known and potential effectors important for plant colonization or infection.

### Xylem Sap-Specific Secreted Proteins Are Dispensable for *V. dahliae ex planta* Development

*Verticillium longisporum* formed a specific secretion response in xylem sap compared to other media showing that the fungus can distinguish between xylem sap and the presence or absence of other plant material. To investigate whether the xylem sap-specific proteins play a major role in fungal colonization of the plant, the top candidates of the protein groups that have been shown to play critical roles in plant colonization (Fradin and Thomma, 2006; Santhanam et al., 2013; Zhang et al., 2017) were analyzed in this study.

Chosen proteins are highlighted in Table 1 with a given name. The follow up genetic analyses of these proteins were conducted with *V. dahliae* JR2 because it can be more easily manipulated genetically compared with the allodiploid *V. longisporum* strain. All candidates from the group “Pathogenicity factors” were included in the follow up experiments. These comprise two NLPs, Nlp2 and Nlp3, and the Cp2 protein. The *V. dahliae* JR2 homolog of *CP1* was tested as well. Cp1 is a virulence factor of *V. dahliae* strain XH-8 in infections on cotton (Zhang et al., 2017). The peptidases that were identified as specifically enriched in xylem sap were named Mep1 and Mep2 and were both included in our genetic analyses. Of the largest group, proteins involved in carbohydrate degradation, the three most highly abundant candidates, the glucoamylase Gla1, the carbohydrate-binding module-containing protein Cbd1, and the α-amylase Amy1, were further investigated.

**FIGURE 3:**
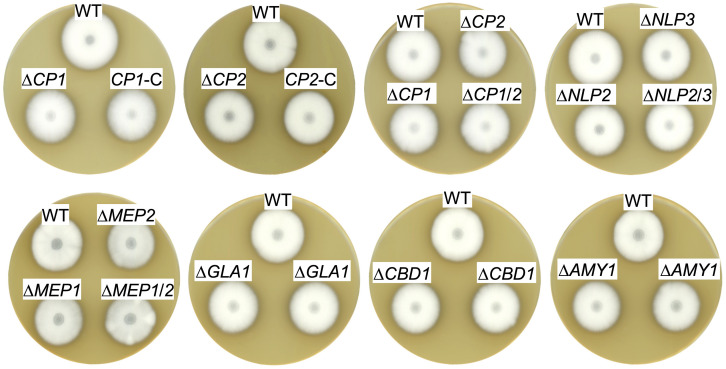
Exoproteins specifically secreted in xylem sap are dispensable for *V. dahliae ex planta* phenotype. The same number of spores of *V. dahliae* JR2 wildtype (WT) and indicated deletion mutant (Δ*CP1*, ΔCP2, Δ*CP1/2*, Δ*NLP2*, Δ*NLP3*, Δ*NLP2/3*, Δ*MEP1*, Δ*MEP2*, Δ*MEP1/2*, Δ*GLA1*, Δ*CBD1*, and Δ*AMY1*) and complementation (*CP1*-C, *CP2*-C) strains were point inoculated on simulated xylem medium (SXM) plates and incubated at 25°C for 10 days. For Δ*AMY1*, Δ*GLA1*, and Δ*CBD1* mutants two transformants were spotted. Top-view scans of the colonies show a similar phenotype of all strains.

For the construction of the corresponding deletion mutants, the open reading frame (ORF) was replaced with either the nourseothricin or hygromycin resistance gene. Correct integration of the deletion cassettes was verified by Southern hybridization (Supplementary Figures S1−S4). To investigate a putative combined effect of proteins in similar groups, the following double deletion strains were constructed as well: *NLP2*/*NLP3*, *MEP1/MEP2* and *CP1/CP2*. Ectopic complementation strains were also constructed for the *CP1* and *CP2* deletion mutants. Phenotypical analysis of all strains revealed no alteration compared to *V. dahliae* JR2 wildtype (WT) growth and development on solid agar plates such as minimal and complete medium, and simulated xylem medium. Additionally, the strains were tested for the involvement in stress responses with at least one stressor tested for each strain. The stress inducing agent was added to minimal medium. The cell wall perturbing agents SDS and ethanol or the oxidative stressor hydrogen peroxide were used. All single deletion, double deletion and complementation strains exhibited a similar morphological development to *V. dahliae* WT, which is exemplified by growth on SXM (Figure 3).

Overall, these results suggest that Cp1, Cp2, Nlp2, Nlp3, Mep1, Mep2, Gla1, Cbd1, and Amy1, that were found to be enriched specifically in xylem sap cultures, are dispensable for vegetative growth, development and stress response of *V. dahliae.*

### Xylem Sap-Specific CAZymes, Metalloproteases, and Cerato-Platanin Proteins Are Dispensable for *V. dahliae* JR2 Pathogenicity in Tomato Infections

**FIGURE 4:**
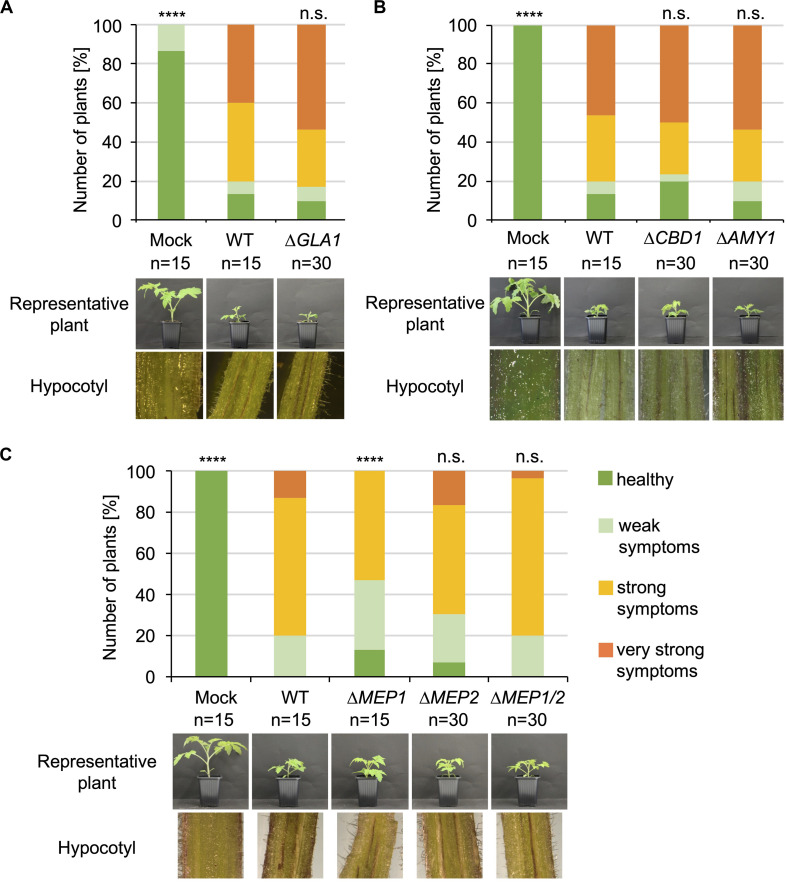
CAZymes and metalloproteases specifically secreted in xylem sap are dispensable for *V. dahliae* pathogenicity on tomato. 10-day-old tomato seedlings were root-infected with spores of *V. dahliae* JR2 (WT) and the indicated single and double deletion strains (Δ*GLA1*, Δ*CBD1*, Δ*AMY1*, Δ*MEP1*, Δ*MEP2*, and Δ*MEP1/2*). Uninfected plants (mock) served as control. The disease index was assessed after 21 days. Representative plants and discolorations of the hypocotyls are shown for each infection. The number (n) of plants is shown for each fungal strain or mock treatment. Significant differences compared to WT are indicated and were calculated with the two-tailed Mann-Whitney Test, *p*-value: ****:*p* < 0.0001, n.s., not significant. **(A)** Infections with *GLA1* (glycoamylase) deletion strains resulted in the same stunting phenotype as WT infections. The standard deviation of the three measured parameters of the plants (plant height, leaf length, and fresh weight) in the four categories ranging from healthy plants to plants with very strong symptoms was on average 13% within one category and did not exceed 30%. **(B)** Strains with deletion of *CBD1* (carbohydrate-binding domain) or *AMY1* (amylase) resulted in a WT-like induction of disease symptoms. The standard deviation in the four categories was on average 9% within one category and did not exceed 27%. **(C)** Absence of metalloprotease Mep1 results in a slightly less virulent infection phenotype compared to WT whereas absence of Mep2 or both metalloproteases exhibit WT-like *V. dahliae* infection behavior on tomato. The standard deviation in the four categories was on average 16% within one category and did not exceed 31%.

**FIGURE 5:**
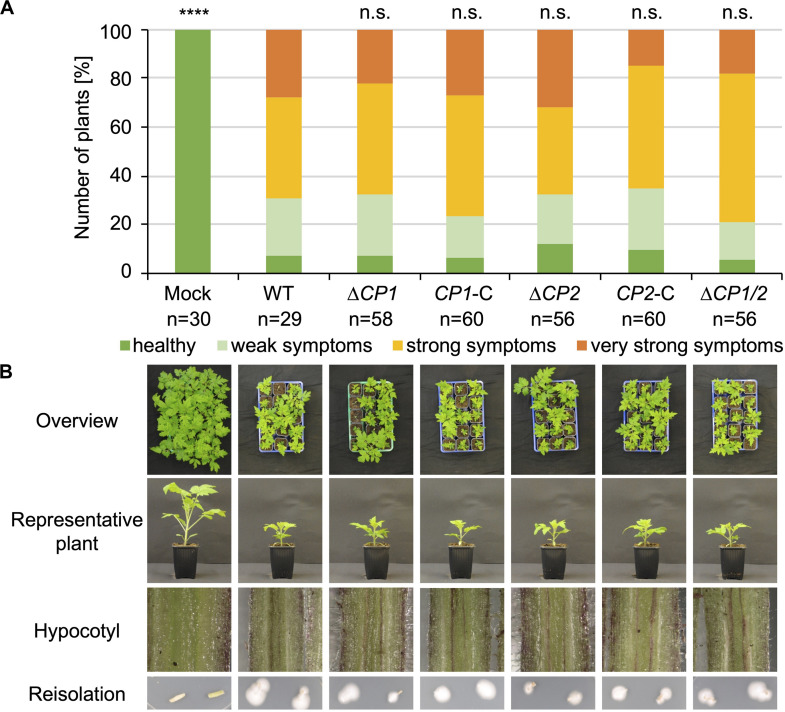
*V. dahliae* Cp1 and Cp2 are dispensable for virulence on tomato. 10-day-old tomato seedlings were infected by root-dipping in a spore suspension of *V. dahliae* JR2 (WT) and the indicated deletion (Δ*CP1*, Δ*CP2*, and Δ*CP1/2*) and complementation strains (*CP1*-C, *CP2*-C). **(A)** The stack diagram shows the percentage of plants with the respective disease index 21 days post-inoculation. The number (n) of treated plants is given for each fungal strain. **(B)** Representative plants, discolorations of the hypocotyls, and the fungal outgrowth of the stems are shown. The experiment was repeated twice. *CP1* and *CP2* deletion strains infect tomato plants to the same extent as WT and the complementation strains. Significant differences compared to WT are indicated and were calculated with the two-tailed Mann-Whitney Test, p-value: ****: *p* < 0.0001, n.s., not significant. The standard deviation of the measured parameters of the plants (plant height, leaf length, and fresh weight) in the four categories was on average 17% within one category and did not exceed 31%.

The functions of the proteins that were specifically enriched after cultivation in xylem sap would be predicted to be important in the interaction with plant substrates in the host xylem sap. Therefore, all *V. dahliae* single and double deletion strains were tested for their virulence on tomato. 10-day-old tomato seedlings were root-inoculated with the indicated mutant strains, and plants treated with demineralized water were used as mock controls. Disease symptoms were measured 3 weeks after inoculation. The stack diagrams display the percentage of plants exhibiting the respective symptoms (Figures 4, 5). We found that plants infected with *GLA1, CBD1, AMY1, MEP2, MEP1/MEP2, CP1, CP2, CP1/CP2* deletion strains or *CP1* or *CP2* complementation strains showed similar disease symptoms as the WT-infected plants. That is, all fungal infections resulted in a similar stunting phenotype as WT colonization, and plant defense reactions were observed by the discoloration of the hypocotyls in all infected plants. Plants infected with the *MEP1* deletion strain show a less severe disease symptom development compared to WT-infected plants whereas the double deletion strain leads to a WT-like infection phenotype. This suggests different functions of the two Mep proteins in virulence. These experiments demonstrate that except Mep1 other tested CAZymes, metalloproteases or cerato-platanin proteins do not affect *V. dahliae* pathogenicity under the tested conditions.

### Nlp3-GFP Is Secreted Into the Extracellular Space

As described above, two NLPs were detected in the xylem sap-specific exoproteome. Members of this group are known to contribute to *V. dahliae* pathogenicity and to exhibit host-specific roles (Wang et al., 2004; Zhou et al., 2012; Santhanam et al., 2013). Nlp1 and Nlp2 contribute to *V. dahliae* JR2 virulence on tomato and *Arabidopsis* (Santhanam et al., 2013), whereas corresponding proteins in *V. dahliae* V592 did not alter virulence on cotton (Zhou et al., 2012). Additionally, Nlp3 did not show any cytotoxic activity on *N. benthamiana* and has not been further characterized (Santhanam et al., 2013). Because our exoproteome approach identified Nlp2 and Nlp3 as specifically secreted in xylem sap, we subsequently carried out additional analyses of the roles of these proteins.

**FIGURE 6:**
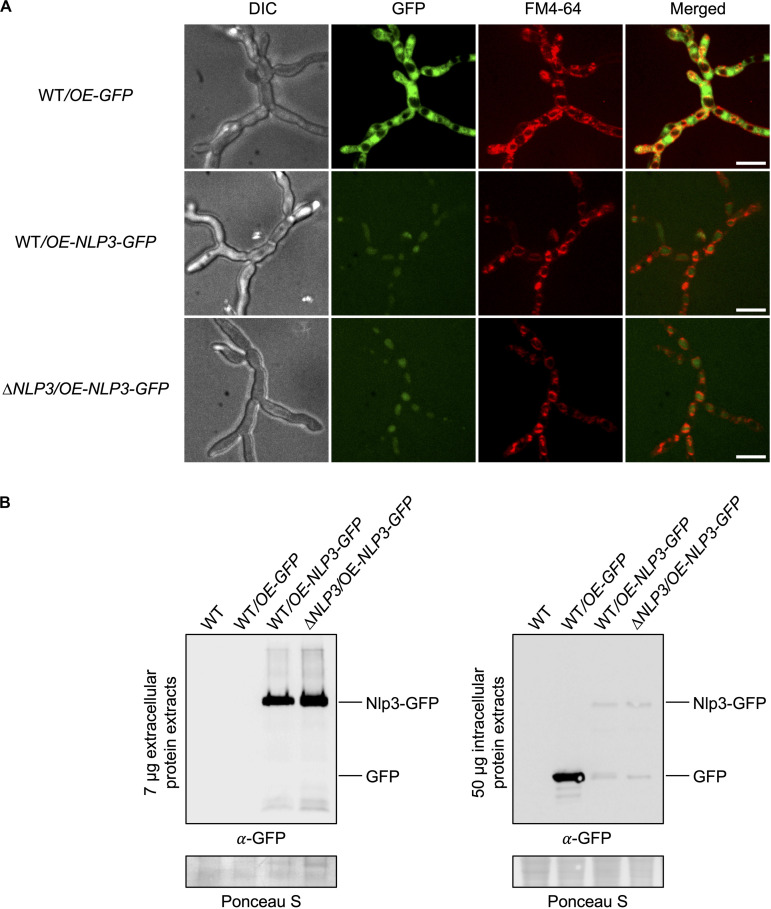
Nlp3-GFP is secreted into the extracellular space. *V. dahliae* JR2 (WT) ectopically overexpressing *GFP* (WT/OE-*GFP*) and WT and *NLP3* deletion strains ectopically overexpressing *NLP3-GFP* (WT/OE-*NLP3-GFP* and Δ*NLP3*/OE-*NLP3-GFP*, respectively) were tested for subcellular localization by fluorescence microscopy in panel **(A)** and localization and production of the intact full-length fusion protein intra- and extracellular by western hybridization in panel **(B)**. **(A)** Confocal microscopy of WT/OE-*NLP3-GFP* and Δ*NLP3*/OE-*NLP3-GFP* strains show the accumulation of the GFP signal inside the red-stained vacuoles whereas the strain WT/OE-*GFP* exhibits GFP signals in the cytoplasm. Spores of the indicated fungal strains were inoculated in 300 μl liquid potato dextrose medium (PDM) in μ-slide 8 well microcopy chambers (Ibidi) and incubated at 25°C overnight. Fungal hyphae were stained with the membrane-selective styryl dye *N*-(3-triethylammoniumpropyl)-4-(*p*-diethylaminophenyl-hexatrienyl) pyridinium dibromide (FM4-64). Scale bar = 10 μm. **(B)** Fungal strains grown in liquid simulated xylem medium (SXM) at 25°C for 24 h. Western hybridization with α-GFP antibody was performed with 7 μg extracellular protein extracts from the culture supernatant and 50 μg intracellular protein extracts. Ponceau S staining served as loading control and WT was used as negative control. Analysis of extracellular proteins in the supernatant of WT/OE-*NLP3-GFP* and Δ*NLP3*/OE-*NLP3-GFP* strains revealed strong signals for Nlp3-GFP with a size of 52 kDa whereas intracellular protein extracts result in faint bands at the size of the fusion protein as well as for free GFP (∼27 kDa). In the control strain WT/OE-*GFP* a strong GFP signal was detected in intracellular extracts.

To monitor the secretion of Nlp3 in liquid media, *V. dahliae* JR2 and *NLP3* deletion strains ectopically overexpressing *NLP3-GFP* were constructed. These strains were confirmed by Southern hybridization (Supplementary Figure S1). Growth characteristics of the strains expressing *NLP3-GFP* were analyzed as described for the other deletion strains. Similar to the deletion strains, the *NLP3-GFP* overexpressing mutants did not show any significant growth variation in comparison to the WT strain under the tested conditions as shown on CDM plates (Supplementary Figure S5). Confocal microscopy of the *NLP3-GFP* strains confirmed the production of a GFP signal derived by the Nlp3-GFP fusion protein with an intracellular location at vacuoles (Figure 6A). The expression and secretion of the fusion protein was analyzed by western experiments using a 24 h-old SXM culture. Intracellular proteins were extracted from fungal mycelium, extracellular proteins were precipitated from the culture supernatant and subjected to SDS−PAGE. Western analysis of the *NLP3-GFP* strain confirmed the overexpression and secretion of the fusion protein in pectin-rich medium (Figure 6B). WT and *NLP3* deletion strains ectopically overexpressing *NLP3-GFP* (WT*/OE-NLP3-GFP* and Δ*NLP3/OE-NLP3-GFP*, respectively) revealed strong signals for Nlp3-GFP with a size of 52 kDa in extracellular extracts whereas intracellular protein extracts result in faint bands at the size of the fusion protein as well as for free GFP (∼27 kDa). These results correspond to the low GFP intensity in the vacuoles (Figure 6A), which may reflect the amount of protein that is not secreted during overexpression and is, therefore, localized to the vacuoles for degradation. For the control strain *V. dahliae* JR2 expressing free GFP (WT/OE-*GFP*), no signal was detected in extracellular space and a strong GFP signal was detected in intracellular extracts. These data corroborate that Nlp3 is primarily a secreted protein and its expression levels neither influence growth nor development.

### Nep1-Like Proteins Nlp2 and Nlp3 Contribute to *V. dahliae* Virulence on Tomato

**FIGURE 7:**
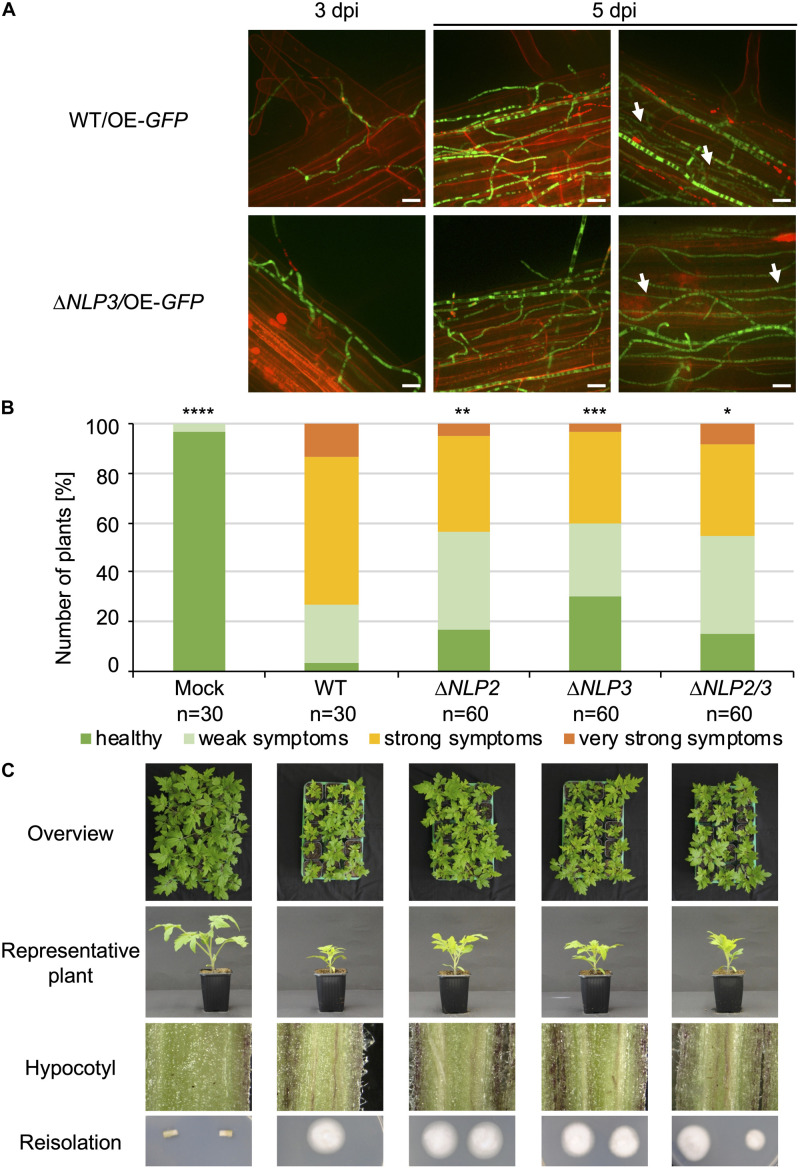
Necrosis and ethylene inducing-like effectors Nlp2 and Nlp3 contribute to *V. dahliae* virulence on tomato. The *NLP3* deletion strain was tested for *A. thaliana* root colonization and single and double deletion strains of *NLP2* and *NLP3* were tested for pathogenicity on tomato. Infected plants were incubated in the climate chamber under 16:8 h light:dark at 22–25°C. **(A)** 3-week-old *A. thaliana* seedlings were root-infected with spores of *V. dahliae* JR2 and *NLP3* deletion strain overexpressing free GFP (WT/OE-*GFP* and Δ*NLP3/*OE-*GFP*, respectively). At 3 and 5 days post-inoculation (dpi) the colonization of the fungal hyphae was monitored with root cells stained by 0.05% propidium iodide/0.01% silwet solution. The experiment was repeated twice with two individual transformants of Δ*NLP3/*OE-*GFP*. Fluorescence microscopy pictures show similar initial colonization of *V. dahliae* WT/OE-*GFP* and Δ*NLP3/*OE-*GFP* on the root surface at 3 dpi and whole root colonization at 5 dpi. White arrows indicate hyphae growing in the xylem cylinder. Scale bar = 10 μm. **(B,C)** 10-day-old tomato seedlings were root-infected with spores of *V. dahliae* JR2 and the indicated single *NLP2* (Δ*NLP2*) and *NLP3* (Δ*NLP3*) and *NLP2/3* double deletion strains (Δ*NLP2/3*). Uninfected plants (mock) served as control. Representative plants, discolorations of the hypocotyls, and the fungal outgrowth of the surface sterilized stems are shown. The disease index was assessed at 21 dpi, which shows that plants infected with *NLP2* and *NLP3* single and double deletion strains exhibit an intermediate phenotype when compared with mock and WT-infected plants. The number of treated plants (n) is shown for each fungal strain. Significant differences compared to WT are indicated and were calculated with the two-tailed Mann-Whitney Test, *p*-values: *: *p* < 0.05, **: *p* < 0.01, ***: *p* < 0.001, and ****: *p* < 0.0001. The standard deviation of the three measured parameters of the plants (plant height, leaf length and fresh weight) in the four categories ranging from healthy plants to plants with very strong symptoms was on average 15% within one category and did not exceed 29%.

Nlp2 only exhibited minor effects in the *V. dahliae*−tomato system (Santhanam et al., 2013) whereas Nlp3 has not been tested for *V. dahliae* pathogenicity. We first analyzed the adherence to the root and further root colonization of the *NLP3* deletion strain on *A. thaliana* with fluorescence microscopy. The *NLP3* deletion strain expressing free *GFP* under the control of the *gpdA* promoter (Δ*NLP3/OE-GFP*) and the WT control overexpressing *GFP* (WT*/OE-GFP*) were used for root inoculation of 3-week-old *Arabidopsis* seedlings. The root colonization at 3 and 5 days post-inoculation was indistinguishable between WT*/OE-GFP* and Δ*NLP3/OE-GFP* (Figure 7A). Initial root colonization was observed at 3 days following inoculation and whole roots were covered with fungal hyphae after 5 days. Both strains were able to colonize the xylem vessels (Figure 7A, white arrows) suggesting that Nlp3 is dispensable for *A. thaliana* root colonization.

Furthermore, the effect on pathogenicity toward tomato was investigated. Tomato infections were carried out as described above. All tested deletion strains were compromised in virulence compared to WT, but the plants nevertheless developed disease symptoms (Figure 7B). All infected plants exhibited stem discolorations and fungal outgrowth was detected from surface sterilized stems (Figure 7C, bottom rows). Symptom development in plants colonized with deletion strains was less severe compared to WT infection. Tray overviews with 15 treated plants, which is a representative number of plants considering fluctuations in the infection success, nicely demonstrate the differences between different strains (Figure 7C, top row). A representative plant also shows the less stunted phenotype of plants treated with *NLP2* and *NLP3* single and double deletion strains in comparison to WT infected plants (Figure 7C, 2nd row). Translating the disease symptoms into the different categories revealed that about 60% of tested plants exhibited no or only mild symptoms compared to approximately 25% of the WT-treated plants (Figure 7B). Infections with the Δ*NLP2*Δ*NLP3* strain resulted in a similar disease index compared to the single deletion strains and, therefore, showed no additive effect of the two deleted *NLP* genes.

In conclusion, the infection study demonstrated that *NLP2* and *NLP3* contribute to *V. dahliae* JR2 virulence on tomato. Deletion of the genes still resulted in induction of disease symptoms suggesting that the fungal strains are well able to penetrate the plant and that Nlp2 and Nlp3 play primarily a role inside of the plant. This is consistent with the exoproteome approach that identified Nlp2 and Nlp3 as xylem sap-specific secretion proteins, in accordance with a role during later infection steps in the xylem vessels. These experiments show that our proteomic approach successfully identified a xylem sap-specific group that includes proteins that are uniquely required in the xylem sap of the plant. While other tested proteins may have redundant functions, we were able to identify NLPs, which are only secreted in a specific environment, as candidates important for *Verticillium* infection.

## Discussion

Fungi require sensing and adapting mechanisms throughout their life cycle. Different environmental cues induce different secretion responses enabling the pathogen to react to changes in e.g., nutrient supply or host defense responses (McCotter et al., 2016).

Our experiments provide evidence for the ability of the allodiploid *V. longisporum* to distinguish between different environments and to induce media-dependent secretion responses. *V. longisporum* secretes a general protein response pattern in various non-plant-related media, which reflects a situation outside of the plant. During cultivation in pectin-rich SXM or plant-extracted xylem sap, the fungus reacts to its surrounding and secretes specific proteins important for the degradation of plant material and the colonization of the xylem. These results imply a complex recognition of plant material in the environment. Further they suggest that SXM lacks the full capacity to mimic the natural growth habitat when compared to our filtered xylem sap.

Xylem sap consists of water, plant defense proteins, hormones and low concentrations of amino acids and sugars that are transported to upper parts of the plant through the transpiration stream (Singh et al., 2010; Carella et al., 2016). The xylem sap is a unique niche for fungal growth due to its low and imbalanced nutrient supply (Singh et al., 2010; Carella et al., 2016). It is important for *Verticillium* species to recognize this specific environment and adapt to it by secreting colonization-related proteins. In prior studies, we demonstrated that *B. napus* xylem sap inhibits growth of *V. longisporum*. No significant changes were observed in fungal protein expression after treatment with uninfected versus infected xylem sap. Cultivation in filtered xylem sap excluding proteins larger than 3 kDa leads to improved fungal growth compared to unfiltered xylem sap. Filtered xylem sap induces higher production of spores, which are required to colonize the host xylem system. These results indicate that the fungus senses signal molecules that induce the adaptation to the environment (Singh et al., 2012). The fungus is able to adapt to the low-nutrient and imbalanced amino acid supply in the xylem sap by activating the cross-pathway (Timpner et al., 2013). In filamentous fungi, this process is controlled by the cross-pathway control transcription factor Cpc1, which is encoded by a homolog of the yeast gene *GCN4* (general control non-derepressed) (Mösch et al., 1991; Hoffmann et al., 2001; Timpner et al., 2013). Knockdowns in *V. longisporum* and knockouts in *V. dahliae* revealed that Cpc1 is required for growth under amino acid starvation conditions and successful colonization of the host plants (Timpner et al., 2013). These findings show that *V. longisporum* senses and reacts to its host environment to survive.

Proteomic data from *in planta* experiments are scarce due to the low fungal protein concentration compared to the plant proteins. Studies with the xylem-colonizing plant pathogen *Fusarium oxysporum* detected few fungal proteins in the tomato xylem sap proteome upon fungal infection. In total, 14 small, cysteine-rich proteins were identified as Six (secreted in xylem) proteins that determine host specificity (Houterman et al., 2007; Lievens et al., 2009; Schmidt et al., 2013). Additionally, secreted enzymes were identified including oxidoreductases, peroxidases and metalloproteases (Houterman et al., 2007; Schmidt et al., 2013). Members of these enzyme classes are also present in our exoproteome data set indicating similar strategies of xylem colonizing fungi. However, most works concentrate on the plant proteins in the xylem proteome and investigate on the differences to mock-treated plants or to symbiotic interactions (Subramanian et al., 2009; Gawehns et al., 2015; de Lamo et al., 2018). SXM was developed to mimic the *in planta* environment, but mainly contains amino acids and the complex carbon source pectin (Neumann and Dobinson, 2003). Pectin is found in plant cell walls where it strengthens the wall integrity (Yadeta and Thomma, 2013). The degradation of this complex branched polysaccharide demands the action of several CAZymes (Yang et al., 2018). This situation is reflected in our SXM-derived exoproteome. In the SXM-specific and the core exoproteome we found 64 and 90 CAZymes, respectively, of which the enzymes acting on pectin are especially overrepresented with 32 and 20 proteins. A study on the *V. dahliae* secretome used minimal medium with cotton root fragments (Zhang et al., 2017). Several secreted proteins were identified including 12 cellulases, five pectate lyases, two chitinases, 13 proteases and one cerato-platanin domain containing protein (Cp1) (Zhang et al., 2017). A number of these proteins were detected in our SXM-specific and core exoproteome. However, no overlap to our xylem sap-specific secretome was detected suggesting that the SXM-induced response of *Verticillium* spp. is more similar to the presence of root fragments. Our data set was also compared with upregulated fungal genes after *V. dahliae* root-inoculation of *A. thaliana* seedlings for 1 day (Scholz et al., 2018). Of these upregulated genes, we identified corresponding proteins in our xylem sap as well as the SXM-derived exoproteomes. Homologs of genes coding for ten out of the 21 *V. dahliae/V. alfalfae* proteins listed in our xylem sap-specific exoproteome (Table 1) overlap with the upregulated *V. dahliae* genes induced after *A. thaliana* root infection (Scholz et al., 2018). This suggests that our xylem sap-specific secretome allows us to pinpoint candidate genes that are expressed *in planta* upon infection and, therefore, represents a technically easier feasible approach for the identification of secreted proteins.

The impact of compatible and incompatible host-pathogen interactions was studies in several studies. The haploid *V. dahliae* responds differently in a susceptible and tolerant olive cultivar (Jiménez-Ruiz et al., 2019). In the susceptible cultivar, the fungus significantly induced expression of genes involved in niche-adaptation, pathogenicity and microsclerotia development (Jiménez-Ruiz et al., 2019). Similarly, the transcriptome of two *V. dahliae* strains with different virulence levels on cotton were analyzed. The strain with reduced virulence exhibited more repressed genes, of which most are related to pathogenesis (Jin et al., 2019). These differences in the host-pathogen interaction indicate that it is possible that the unfiltered xylem sap proteins could induce a more fine-tuned secretion response of the fungus and comparison to xylem sap of infected plants will be interesting, however, the comparison to other studies shows that our filtered xylem sap is closer to mimic the natural plant habitat compared to SXM. Overall, these results corroborate that the fungus senses changes in its environment and responds with different secretion patterns.

In our approach, we focused on the set of proteins that contain a signal peptide and are predicted to be secreted as effector proteins of filamentous fungi, these are typically exported via the conventional Golgi-dependent secretory route (de Jonge et al., 2010; Wang et al., 2018). This allowed the exclusion of putative contaminating intracellular proteins from lysed fungal cells in the extracellular medium. In addition, a number of putatively secreted proteins was detected in *V. longisporum* culture supernatants after cultivation in SXM and xylem sap (Supplementary Tables S2, S3). These may represent unconventionally secreted proteins. The xylem sap proteome is enriched in unconventionally secreted proteins upon infection with pathogens as *Fusarium* with tomato as host (Gawehns et al., 2015; de Lamo et al., 2018). The formation of extracellular vesicles derived from *Fusarium oxysporum* f. sp. *vasinfectum* induces a phytotoxic response in plants (Bleackley et al., 2020). Studies on the *V. dahliae* exoproteome induced by cotton-containing medium support that *V. dahliae* also has other means of exporting proteins than the conventional secretory pathway (Chen et al., 2016; Wang et al., 2018). These unconventional secretion systems are Golgi-independent and include the export of proteins without signal peptides (Wang et al., 2018). Concluding, there are different pathways for protein secretion that allow the export control of different protein sets that impact the host-pathogen outcome. They include conventionally as well as non-conventionally secreted proteins and the knowledge on how these different systems are regulated and coordinated is still lacking.

Another tight and fast adapting control mechanism of gene expression lies in chromatin modifications, which can be induced by environmental changes (Seidl et al., 2016). Such an epigenetic-mediated control has been observed for effector expression in *Leptosphaeria maculans.* Effector genes often reside in AT-rich regions of the genome. These are associated with heterochromatin and explain the silenced state of effector expression. Upon leaf infection chromatin-mediated repression is abolished and gene expression is upregulated in *L. maculans* (Soyer et al., 2014; Tan and Oliver, 2017). As *V. longisporum* responds to the presence of plant-related compounds by inducing specific exoproteome patterns, it will be interesting to shed light on a putative chromatin modification contribution to these unique responses.

Transcriptional regulators can induce the gene expression of several effectors at once. For example, the transcription factors Som1, Vta2 and Vta3, that are required for sequential steps of infection, control similar but also distinct sets of secreted proteins involved in virulence (Tran et al., 2014; Bui et al., 2019). All three proteins are involved in the regulation of, for example, *NLP2* (Tran et al., 2014; Bui et al., 2019). The expression of the two cytotoxic NLPs, *NLP1* and *NLP2*, from *V. dahliae* has been analyzed during host colonization. When colonizing tomato plants, both transcript levels were elevated although only *NLP1* expression was increased during colonization of tobacco plants. The *in planta* expression of *NLP1* and *NLP2* corresponds to the infection phenotype of the deletion strains (Santhanam et al., 2013). These results confirm the hypothesis of a sensitive control mechanism. It shows that effectors may act host-specifically and are only expressed in suitable hosts supporting the idea of a fine-tuned response.

NLPs can have a cytotoxic effect, which is induced upon binding to plant sphingolipid glycosylinositol phosphorylceramides (Lenarčič et al., 2019). In this study, we identified two NLPs, Nlp2 and Nlp3, as effectors specifically secreted in xylem sap. The *NLP2* single deletion strain was shown previously to contribute to *V. dahliae* virulence (Santhanam et al., 2013). Single as well as double deletions of the corresponding genes resulted in compromised pathogenicity on tomato. Tomato infections with the Δ*NLP2*Δ*NLP3* strain resulted in a similar disease index compared to the single deletion strains. In contrast, the lack of two metalloproteases of *Fusarium oxysporum* f. sp. *lycopersici* result in additive effects on pathogenicity. They presumably act on different target sites and together achieve the inactivation of the plant defense protein (Jashni et al., 2015). It is not yet clear why the lack of *V. dahliae* Nlp2 or Nlp3 results in the same effect as the lack of both proteins and it remains to be elucidated whether they compete for binding the same plant receptor to induce plasma membrane leakage. The study on Nlp1 and Nlp2 showed that the *in planta* expression profile of the proteins needs to be timely coordinated to influence the pathogenicity phenotype (Santhanam et al., 2013). Therefore, it remains to be shown whether presence or absence of Nlp2 or Nlp3 affect differential expression of the corresponding proteins.

Other xylem sap-specifically enriched candidates did not show an effect on *V. dahliae* pathogenicity on tomato. This may be due to redundant or greater contributions of the hundreds of secreted *Verticillium* effectors (Klosterman et al., 2011). Our approach revealed a high number of CAZymes among the *V. longisporum* secretome representing 171 out of the total 396 identified proteins (Supplementary Table S6). Overall, the *V. dahliae* genome exhibits a strikingly high repertoire of CAZymes, especially pectin-degrading enzymes (Klosterman et al., 2011). This suggests that proteins with redundant functions are secreted and explains the WT-like infections of *V. dahliae* strains lacking one of the tested CAZymes (glucoamylase Gla1, putative polysaccharide mono-oxygenase Cbd1 and α-amylase Amy1).

We identified two metalloproteases, Mep1 and Mep2, belonging to two different groups of metalloproteases (M36 and M43, respectively) as xylem sap-specific secreted proteins. Tomato infections with *V. dahliae MEP1* deletion strain result in less severe disease symptoms compared to WT infections indicating that Mep1 contributes to *V. dahliae* pathogenicity. The Δ*MEP1*Δ*MEP2* double deletion strain infected the tomato plants to the same extend as the WT, which suggest that loss of Mep2 compensates the loss of Mep1 and both enzymes fulfill different yet unknown functions in virulence. Metalloproteases promote fungal virulence by degrading host proteins (Brouta et al., 2002; Jashni et al., 2015). Plant chitinases degrade chitin of the fungus, which elicits the plant defense response (Naumann and Price, 2012). *V. dahliae* possesses the ability to truncate extracellular chitin-binding domain-containing chitinases (Jashni et al., 2015; Han et al., 2019). The *V. dahliae* proteome comprises two M43 and six M36 peptidases implicating that other metallopeptidases are able to complement the absence of Mep1 and Mep2. Furthermore, synergistic actions of metallo- and serine proteases have been reported in *F. oxysporum* (Jashni et al., 2015), providing more evidence for the hypothesis of functional redundancy.

We also investigated on the cerato-platanin domain containing proteins Cp1 and Cp2. Cp1 was first identified in the exoproteome of the *V. dahliae* strain XH-8 when incubated in minimal medium supplemented with cotton root fragments. In this system, Cp1 was required for cotton virulence and is suggested to function as a chitin scavenger to prevent fungal recognition by the plant (Zhang et al., 2017). We detected Cp1 in our core exoproteome and included it in our study to investigate on putative synergistic actions of the two present in the *V. dahliae* genome. Our results show that Cp1 and Cp2 are dispensable for *V. dahliae* pathogenicity on tomato. These results indicate that effectors may have strain- and host-specific activities.

To our knowledge, our proteomic study is the first report identifying the differences between the secretion responses of *V. longisporum* in host xylem sap, the xylem sap mimic SXM and other media. Non-plant related environments elicited a similar broad exoproteome pattern whereas the plant-related media, SXM and xylem sap, induced similar but also distinct responses. These results indicated that the fungus has the capacity to sense differences in the presence of plant-related compounds and therefore rules out SXM as xylem sap mimic. Additionally, our approach identified necrosis and ethylene inducing-like proteins Nlp2 and Nlp3 in the xylem sap-specific secretome. These proteins are required for *V. dahliae* pathogenicity with roles in later steps of infection and display potential targets for control strategies of Verticillium wilt.

## Materials and Methods

### Fungal Strains and Growth Conditions

*Verticillium* strains (Supplementary Table S7) were cultivated in liquid SXM modified from Neumann and Dobinson (2003) as described by Hollensteiner et al. (2017) for conidiospore formation and in liquid potato dextrose medium [Potato Dextrose broth (Carl Roth)] for mycelial growth. Cultures were incubated at 25°C under constant agitation at 115−125 rpm. For long-term storage spores were maintained in closed vials with 25% glycerol at −80°C.

For the exoproteome comparison, *V. longisporum* 43 (Vl43) was inoculated with 1.5 × 10^6^ spores per 150 ml potato dextrose medium and incubated for 4 days at 25°C and 150 rpm agitation. Each culture was centrifuged and the mycelium and spore sediment was resuspended in 150 ml extracted xylem sap of *B. napus*; SXM, the minimal medium Czapek-Dox medium (CDM, modified from Smith, 1949) supplemented with 7% extracted xylem sap or plant proteins; H_2_O and H_2_O supplemented with 0.1% glucose; YNB (yeast nitrogen base: 1.5 g/l YNB, 5 g/l (NH_4_)_2_SO4, 20 g/l glucose, ad 1 l H_2_O) and 10% vegetable juice (V8, Campbell Soup Company, Sexton et al., 2009). After an additional incubation period of 4 days, proteins of the supernatant were precipitated with TCA/acetone.

### Xylem Sap Extraction

Xylem sap was extracted from *B. napus* (Falcon, Norddeutsche Pflanzenzucht). Seeds were surface sterilized with 70% ethanol and sown on sand. Plants were grown at long-day condition (16 h light: 8 h dark) and 22°C. 7-day-old seedlings were transferred into a soil/sand (1:1) mixture and grown for 42 days. To extract the xylem sap, plants were cut at the height of the first internode and the xylem sap was collected. The xylem sap was filtered through Vivaspin 15R centrifugal concentrators (Sartorius) with a molecular weight cut off of 10 kDa, allowing smaller plant molecules to pass through and excluding larger molecules. This filtered xylem sap was directly used as medium for inoculation. For one experiment, the xylem sap of 500 plants was extracted resulting in approximately 500 ml of pure xylem sap.

### Genomic DNA Extraction

For isolation of genomic DNA (gDNA) from fungal powder, the method modified from Kolar et al. (1988) was used. The fine powder was mixed with 800 μl of lysis buffer [50 mM Tris (pH 7.5), 50 mM EDTA (pH 8), 3% (w/v) SDS and 1% (v/v) ß-mercaptoethanol] and incubated at 65°C for 1 h. Before the mixture was centrifuged for 20 min at 13000 rpm, 800 μl phenol were added. The upper aqueous phase was transferred to a new tube. To denature the proteins, 500 μl chloroform were added, mixed and centrifuged for 10 min at 13000 rpm. The upper phase was mixed with 400 μl of isopropanol for precipitation of gDNA and centrifuged for 2 min at 13000 rpm. The sedimented gDNA was washed with 70% (v/v) ethanol. The gDNA was dried at 65°C for approximately 25 min before it was dissolved in up to 100 μl deionized H_2_O containing 2 μl RNase A (10 mg/ml) and treated at 65°C for 30 min to remove RNA.

### Plasmid and Strain Construction

The desired genes and flanking regions for plasmid construction were amplified of *V. dahliae* JR2 WT gDNA with the Phusion High Fidelity Polymerase, *Taq* DNA Polymerase (both Thermo Fisher Scientific) or Q5 High Fidelity Polymerase (New England Biolabs). Primers are listed in Supplementary Table S8.

GeneArt Seamless Cloning and Assembly Kit (Thermo Fisher Scientific) was used for the cloning strategy. Plasmids are listed in Supplementary Table S9. *E. coli* strain DH5α was utilized for cloning reactions and propagation of plasmids. Transformation of *E. coli* was performed based on a heat shock method (Inoue et al., 1990). *A. tumefaciens* AGL-1 cells were transformed with the desired plasmids via a freeze-thaw method (Jyothishwaran et al., 2007) and was then utilized for an *A. tumefaciens* mediated transformation of *V. dahliae* spores, which was performed based on the method described by Mullins et al. (2001). Details on specific mutant strains in haploid *V. dahliae* are given in Supplementary Text S1.

### Southern Hybridization Analysis

For verification of *V. dahliae* deletion strains, the corresponding flanking region of the gene was amplified and labeled as probe. Genomic DNAs were restricted with indicated enzymes overnight (Supplementary Figures S1−S4). The mixture was separated on a 1% agarose gel and DNA was transferred to a Hybond-N membrane (GE Healthcare) by blotting. DNA on the membrane was hybridized overnight to the probe. CDP-Star Detection reagent (GE Healthcare) was used to detect chemiluminescence signals according to the manufacturer’s instructions.

### Protein Assays and Western Hybridization Analysis

Extracellular proteins from the supernatant of SXM cultures were precipitated with 10% TCA (w/v) in acetone at 4°C overnight. This mixture was centrifuged at 4 000 rpm for 60 min at 4°C. The protein sediment was washed three times with 80% (v/v) acetone, once with 100% (v/v) acetone and then dissolved in 8 M urea/2 M thiourea. Intracellular proteins were extracted from ground mycelium with extraction buffer [300 mM NaCl, 100 mM Tris−HCl pH 7.5, 10% glycerol, 1 mM EDTA, 0.02% NP-40, 2 mM DTT and complete EDTA-free protease inhibitor cocktail (Roche)]. Samples were centrifuged for 20 min at 13 000 rpm at 4°C and the supernatants were transferred into fresh test tubes. Protein concentrations were determined using a Bradford-based Roti-Quant assay (Carl Roth). Protein samples were separated in 12% SDS−PAGE gels, followed by protein transfer onto an Amersham Protran 0.45 μm nitrocellulose membrane (GE Healthcare). The membrane was blocked in 5% (w/v) skim milk powder in TBS-T [10 mM Tris−HCl (pH 8), 150 mM NaCl, 0.05% (w/v) Tween 20] and probed with α-GFP antibody (Santa Cruz Biotechnology). As secondary antibody the horseradish peroxidase-coupled α-mouse antibody (115-035-003, Jackson ImmunoResearch) was applied. Detection of chemiluminescent signals was conducted with horseradish peroxidase substrate luminol based chemiluminescence.

### Tryptic Digestion, Mass Spectrometry Analysis, and Protein Identification

For one-dimensional gel analysis, 30 μg of the extracellular protein extract was separated by 12% SDS−PAGE gels. The polyacrylamide gels were incubated 1h in fixing solution (40% (v/v) ethanol, 10% (v/v) acetic acid) and washed twice for 20 min with H_2_O. Gels were colloidal Coomassie stained [0.12% (w/v) CBB G-250, 5% (w/v) aluminum sulfate-(14−18)-hydrate, 10% (v/v) methanol, 2% (v/v) orthophosphoric acid (85%)]. One lane, which derived from one individual culture, was cut into ten pieces of equal size. The excised polyacrylamide gels were in-gel digested with trypsin (Shevchenko et al., 1996). Resulting tryptic peptide mixtures were separated by a reversed-phase liquid chromatographic column (Acclaim PepMap RSLC column, 75 μm × 15 cm, C18, 3 μm, 100 Å, P/N164534, Thermo Fisher Scientific) to further reduce sample complexity prior to mass analyses with an LTQ Velos Pro mass spectrometer (Thermo Fisher Scientific). To identify matches of the detected peptides, an in-house genome-wide protein sequence database of *V. longisporum* was used of which the protein sequences are given in Supplementary Tables S1−S3. Analysis was performed by a Thermo Proteome Discoverer (version 1.3) workflow that integrates Sequest and Mascot search engines. For the search an initial precursor mass tolerance of 10 ppm and fragment mass tolerance of 0.8 Da Carbamidomethylcysteine was used as fixed modification. Oxidized methionine was included as variable modification and two missed cleavages were allowed for each peptide. For peptide and protein validation, a 0.5% false discovery rate was set and determined by using peptide validator with a reverse decoy database. The mass spectrometry proteomics data have been deposited to the ProteomeXchange Consortium via the PRIDE (Perez-Riverol et al., 2019) partner repository with the dataset identifier PXD018533.

Resulting lists of identified proteins were semi-quantitatively processed using the MarVis-Suite software (Kaever et al., 2012). To achieve a broad overview of the total secretome, a threshold of at least two peptides in at least two independent cultivations was set as criteria. Only proteins with a WoLF PSORT extracellular localization prediction of above 12 were considered for further characterization (Horton et al., 2007). The principle component analysis plot of the exoproteomes based on the spectral counts was performed with MarVis-Suite software, and each dot represents one independent culture (Meinicke et al., 2008). Experiments were conducted with at least four cultures with all tested growth conditions. Comparison of the SXM and xylem sap-specific exoproteomes revealed a group of 441 proteins of which the protein sequences and details on their abundance as measured by identified peptides are given in Supplementary Table S1. The signal-to-level (s/l) ratio (see MarVis-Suite handbook^1^) was calculated using as signal the difference between SXM and xylem sap condition averages (or vice versa) and as level the corresponding maximum. Polypeptides with an s/l ratio above 0.3 were considered as candidates with higher intensities in the specific medium and therefore belong to the specifically enriched proteins. Xylem sap-specific proteins are depicted in green and SXM-specific proteins in red. Whereas an s/l ratio below 0.3 was considered not to be specifically enriched and formed the shared core proteome.

The list of proteins was further analyzed and compared to the Ensembl Fungi annotations which are more robust. Candidates with no proper predictions in our preliminary genome-wide protein sequence database of *V. longisporum* (e.g., stop codons in protein sequences, two genes annotated as one) were revealed by comparison with corresponding *V. dahliae* JR2 and *V. alfalfae* VaMs.102 sequences and eliminated from the list. Further analyses are based on the *V. dahliae* JR2 or the *V. alfalfae* VaMs.102 protein sequences (Kersey et al., 2018). Domain predictions and associated families were obtained with InterProScan (Jones et al., 2014) and classification of CAZyme families according to the CAZy database^2^ was specified with dbCAN2 (Zhang et al., 2018) to address putative functions of the proteins. Proteins were considered as putatively secreted with at least one predicted signal peptide by InterProScan or SignalP-5.0 (Almagro Armenteros et al., 2019) or as long as it passed the threshold of 12 as determined by WoLF PSORT (Horton et al., 2007) for the *V. longisporum* 43, *V. dahliae* JR2, or *V. alfalfae* VaMs.102 protein sequence. All details are given in Supplementary Table S4.

### *A. thaliana* Root Infection Assay

*A. thaliana* (Col-0, N1902; Nottingham Arabidopsis Stock Centre) seedlings were inoculated by root-dipping with a conidia suspension of *V. dahliae* JR2 and *NLP3* deletion strain overexpressing *GFP* (1 × 10^7^ spores/ml) based on the method described by Bui et al. (2019). 3-week-old seedlings were used for infection. The roots were incubated in spore solutions with 100 000 spores/ml for 35 min. The plates were further incubated in the plant chamber at long day conditions (22−25°C) and colonization on the roots was monitored at indicated time points. The root was incubated in a staining solution (0.0025% (v/v) propidium iodide, 0.005% (v/v) silwet) for 5 min in the dark. Images of infected roots were taken with a Zeiss Observer Z1 microscope equipped with CSU-X1 A1 confocal scanner unit (Yokogawa), QuandtEM:512SC (Photometrics) digital camera and Slidebook 5.0 software package (Intelligent Imaging Innovations).

### Tomato Infection Assay

*Solanum lycopersicum* (Moneymaker, Bruno Nebelung Kiepenkerl-Pflanzenzüchtung) seeds were surface sterilized with 70% (v/v) EtOH, 0.05% Tween 20, sown on sand/soil (1:1) mixture (Dorsilit, Archut). Plants were grown under a photoperiod of 16 h light and 8 h of darkness at 25 and 22°C, respectively. The tomato pathogenicity assays were performed on 10-day-old *S. lycopersicum* seedlings. The plants were root-inoculated by incubating the roots in 50 ml of 10^7^ spores/ml for 40 min under constant agitation at ∼35 rpm. Mock control plants were treated similarly with dH_2_O.

The seedlings were transferred to pots containing a sand/soil (1:1) mixture and 3 000 000 spores or 3 ml dH_2_O for mock plants were added to the soil. For each strain or control 15 plants were infected. Plants were incubated in the climate chamber at long day conditions for another 21 days before disease symptoms were scored. The fresh weight of the aerial parts, the length of the longest leaf and the height of the vegetation point were measured. These parameters were calculated into a disease score ranking relative to uninfected mock plants. The mean values of mock plants of each parameter were set to 100%. All values above 80% were assigned as “healthy,” 60−80% as “mild symptoms,” 40−80% as “strong symptoms,” lower than 40% as “very strong symptoms” and dead plants as “dead.” The scores for each strain were visualized in a stack diagram displaying the number of plants per disease score relative to the total amount of tested plants from all experiments. As another measure the discoloration of the tomato hypocotyl was observed with a binocular microscope SZX12-ILLB2-200 from Olympus. All treated plants were tested for fungal outgrowth 21 days after infection. The tomato stems were surface sterilized in 70% ethanol, followed by 6% hypochlorite solution each for 8 min before two washing steps with dH_2_O. Stem ends were removed and slices were placed on potato dextrose medium (PDM) plates containing 100 μg/ml chloramphenicol. After incubation of 7 days at 25°C the fungal outgrowth was observed. The pathogenicity assay was performed once with the metalloprotease constructs and once with Δ*GLA1*, Δ*CBD1* and Δ*AMY1* strains. Pathogenicity assays with the *CP* and *NLP* constructs were repeated twice with similar outcome. Significant differences compared to WT were calculated with the two-tailed Mann-Whitney Test (Supplementary Table S10). For each assay 15 plants were infected with WT and 15 plants were used as mock control. Per transformant 15 plants were infected and scores of identical strains were taken together of two individual transformants.

### Accession Numbers and Data Availability

Sequence data for *V. dahliae* were retrieved from Ensembl Fungi with the following accession numbers: *NLP2* (*VDAG_JR2_Chr2g05460a*), *NLP3* (*VDAG_JR2_Chr4g05950a*), *CP1* (*VDAG_JR2_Chr7g00860a*), *CP2* (*VDAG_JR2_Chr2g070 00a*), *MEP1* (VDAG_JR2_Chr8g09760a), *MEP2* (VDAG_JR2_ Chr1g21900a), *GLA1* (*VDAG_JR2_Chr8g11020a*), *CBD1* (*VDAG _JR2_Chr4g04440a*), and *AMY1* (*VDAG_JR2_Chr7g03330a*). Protein sequences for *V. longisporum* are given in Supplementary Table S1 as retrieved from an in-house database or taken from Ensembl Fungi. The mass spectrometry proteomics data are available via ProteomeXchange https://www.ebi.ac.uk/pride/login with identifier PXD018533.

## Data Availability Statement

The datasets presented in this study can be found in online repositories. The names of the repository/repositories and accession number(s) can be found below: https://www.ebi.ac.uk/pride/archive/, PXD018533.

## Author Contributions

MiL, AnK, RH, HK, SB-S, JK, and GB designed and conceived the study. MiL, AnK, IM, AN, and JS performed and analyzed the experiments. MiL, AnK, RH, IM, AN, HK, OV, KF, AlK, MaL, BM, DB, MH, and GB carried out the formal analysis. RH, HK, OV, KF, IF, BM, DB, MH, JK, and GB supervised the whole project. MiL, AnK, RH, and GB wrote the manuscript. All authors interpreted the results, revised, and approved the final manuscript.

## Conflict of Interest

The authors declare that the research was conducted in the absence of any commercial or financial relationships that could be construed as a potential conflict of interest.

## Footnotes

1 http://marvis.gobics.de

2 http://www.cazy.org

## Supplementary Material

FIGURE S1 Verification of *V. dahliae NLP2* and *NLP3* deletion or overexpression constructs.

FIGURE S2 Verification of *V. dahliae CP1* and *CP2* deletion and complementation strains.

FIGURE S3 Verification of *V. dahliae MEP1* and *MEP2* deletion strains.

FIGURE S4 Verification of *V. dahliae GLA1*, *CBD1*, and *AMY1* deletion strains.

FIGURE S5 Expression of *NLP3-GFP* under control of a constitutively active promoter allows *V. dahliae* wildtype-like growth on solid media.

TABLE S1 List of identified proteins in SXM and xylem sap.

TABLE S2 List of other identified proteins without predicted secretion in xylem sap.

TABLE S3 List of other identified proteins without predicted secretion in SXM.

TABLE S4 Annotation of identified proteins in SXM and xylem sap.

TABLE S5 Functional groups of secreted proteins.

TABLE S6 CAZyme classification of secreted proteins.

TABLE S7 *Verticillium* strains used in this study.

TABLE S8 Primers used in this study.

TABLE S9 Plasmids used in this study.

TABLE S10 Mann–Whitney Test.

## References

[B1] Almagro Armenteros J. J. Tsirigos K. D. Sønderby C. K. Petersen T. N. Winther O. Brunak S. (2019). SignalP 5.0 improves signal peptide predictions using deep neural networks. *Nat. Biotechnol.* 37 420–423. 10.1038/s41587-019-0036-z 30778233

[B2] Bleackley M. R. Samuel M. Garcia-Ceron D. McKenna J. A. Lowe R. G. T. Pathan M. (2020). Extracellular vesicles from the cotton pathogen *Fusarium oxysporum* f. sp. *vasinfectum* induce a phytotoxic response in plants. *Front. Plant Sci.* 10:1610. 10.3389/fpls.2019.01610 31998330 PMC6965325

[B3] Brito N. Espino J. J. González C. (2006). The endo-beta-1,4-xylanase xyn11A is required for virulence in *Botrytis cinerea*. *Mol. Plant Microbe Interact.* 19 25–32. 16404950 10.1094/MPMI-19-0025

[B4] Brouta F. Descamps F. Monod M. Vermout S. Losson B. Mignon B. (2002). Secreted metalloprotease gene family of *Microsporum canis*. *Infect. Immun.* 70 5676–5683. 12228297 10.1128/IAI.70.10.5676-5683.2002 PMC128366

[B5] Bui T. Harting R. Braus−Stromeyer S. A. Tran V. Leonard M. Höfer A. (2019). *Verticillium dahliae* transcription factors Som1 and Vta3 control microsclerotia formation and sequential steps of plant root penetration and colonisation to induce disease. *New Phytol.* 221 2138–2159. 10.1111/nph.15514 30290010

[B6] Carella P. Wilson D. C. Kempthorne C. J. Cameron R. K. (2016). Vascular sap proteomics: providing insight into long-distance signaling during stress. *Front. Plant Sci.* 7:651. 10.3389/fpls.2016.00651 27242852 PMC4863880

[B7] Chen J.-Y. Xiao H.-L. Gui Y.-J. Zhang D.-D. Li L. Bao Y.-M. (2016). Characterization of the *Verticillium dahliae* exoproteome involves in pathogenicity from cotton-containing medium. *Front. Microbiol.* 7:1709. 10.3389/fmicb.2016.01709 27840627 PMC5083787

[B8] de Boer A. H. Volkov V. (2003). Logistics of water and salt transport through the plant: structure and functioning of the xylem. *Plant Cell Environ.* 26 87–101. 10.1046/j.1365-3040.2003.00930.x

[B9] de Jonge R. van Esse H. P. Kombrink A. Shinya T. Desaki Y. Bours R. (2010). Conserved fungal LysM effector Ecp6 prevents chitin-triggered immunity in plants. *Science* 329 953–955. 10.1126/science.1190859 20724636

[B10] de Lamo F. J. Constantin M. E. Fresno D. H. Boeren S. Rep M. Takken F. L. W. (2018). Xylem sap proteomics reveals distinct differences between R gene- and endophyte-mediated resistance against Fusarium wilt disease in tomato. *Front. Microbiol.* 9:2977. 10.3389/fmicb.2018.02977 30564219 PMC6288350

[B11] Dodds P. N. Rathjen J. P. (2010). Plant immunity: towards an integrated view of plant–pathogen interactions. *Nat. Rev. Genet.* 11 539–548. 10.1038/nrg2812 20585331

[B12] Dunker S. Keunecke H. Steinbach P. von Tiedemann A. (2008). Impact of *Verticillium longisporum* on yield and morphology of winter oilseed rape (*Brassica napus*) in relation to systemic spread in the plant. *J. Phytopathol.* 156 698–707. 10.1111/j.1439-0434.2008.01429.x

[B13] Eynck C. Koopmann B. Grunewaldt-Stoecker G. Karlovsky P. von Tiedemann A. (2007). Differential interactions of *Verticillium longisporum* and *V. dahliae* with *Brassica napus* detected with molecular and histological techniques. *Eur. J. Plant Pathol.* 118 259–274. 10.1007/s10658-007-9144-6

[B14] Floerl S. Druebert C. Majcherczyk A. Karlovsky P. Kües U. Polle A. (2008). Defence reactions in the apoplastic proteome of oilseed rape (*Brassica napus* var. *napus*) attenuate *Verticillium longisporum* growth but not disease symptoms. *BMC Plant Biol.* 8:129. 10.1186/1471-2229-8-129 19094241 PMC2644697

[B15] Fradin E. F. Thomma B. P. H. J. (2006). Physiology and molecular aspects of Verticillium wilt diseases caused by *V. dahliae* and *V. albo-atrum*. *Mol. Plant Pathol.* 7 71–86. 10.1111/j.1364-3703.2006.00323.x 20507429

[B16] Gawehns F. Ma L. Bruning O. Hellmann H. A. Wilkerson C. G. Kleffmann T. (2015). The effector repertoire of *Fusarium oxysporum* determines the tomato xylem proteome composition following infection. *Front. Plant Sci.* 6:967. 10.3389/fpls.2015.00967 26583031 PMC4631825

[B17] Gijzen M. Nürnberger T. (2006). Nep1-like proteins from plant pathogens: recruitment and diversification of the NPP1 domain across taxa. *Phytochemistry* 67 1800–1807. 10.1016/j.phytochem.2005.12.008 16430931

[B18] Gui Y. J. Chen J. Y. Zhang D. D. Li N. Y. Li T. G. Zhang W. Q. (2017). *Verticillium dahliae* manipulates plant immunity by glycoside hydrolase 12 proteins in conjunction with carbohydrate-binding module 1. *Environ. Microbiol.* 19 1914–1932. 10.1111/1462-2920.13695 28205292

[B19] Han L. B. Li Y. B. Wang F. X. Wang W. Y. Liu J. Wu J. H. (2019). The cotton apoplastic protein CRR1 stabilizes chitinase 28 to facilitate defense against the fungal pathogen *Verticillium dahliae*. *Plant Cell* 31 520–536. 10.1105/tpc.18.00390 30651348 PMC6447012

[B20] Hoffmann B. Valerius O. Andermann M. Braus G. H. (2001). Transcriptional autoregulation and inhibition of mRNA translation of amino acid regulator gene *cpcA* of filamentous fungus *Aspergillus nidulans*. *Mol. Biol. Cell* 12 2846–2857. 10.1091/mbc.12.9.2846 11553722 PMC59718

[B21] Hollensteiner J. Wemheuer F. Harting R. Kolarzyk A. M. Diaz Valerio S. M. Poehlein A. (2017). *Bacillus thuringiensis* and *Bacillus weihenstephanensis* inhibit the growth of phytopathogenic *Verticillium* species. *Front. Microbiol.* 7:2171. 10.3389/fmicb.2016.02171 28149292 PMC5241308

[B22] Horton P. Park K. J. Obayashi T. Fujita N. Harada H. Adams-Collier C. J. (2007). WoLF PSORT: protein localization predictor. *Nucleic Acids Res.* 35 W585–W587. 10.1093/nar/gkm259 17517783 PMC1933216

[B23] Houterman P. M. Speijer D. Dekker H. L. De Koster C. G. Cornelissen B. J. C. Rep M. (2007). The mixed xylem sap proteome of *Fusarium oxysporum*-infected tomato plants. *Mol. Plant Pathol.* 8 215–221. 10.1111/j.1364-3703.2007.00384.x 20507493

[B24] Inderbitzin P. Subbarao K. V. (2014). *Verticillium* systematics and evolution: how confusion impedes *Verticillium* wilt management and how to resolve it. *Phytopathology* 104 564–574. 10.1094/PHYTO-11-13-0315-IA 24548214

[B25] Inoue H. Nojima H. Okayama H. (1990). High efficiency transformation of *Escherichia coli* with plasmids. *Gene* 96 23–28. 10.1016/0378-1119(90)90336-P 2265755

[B26] Isshiki A. Akimitsu K. Yamamoto M. Yamamoto H. (2001). Endopolygalacturonase is essential for citrus black rot caused by *Alternaria citri* but not brown spot caused by *Alternaria alternata*. *Mol. Plant Microbe Interact.* 14 749–757. 10.1094/MPMI.2001.14.6.749 11386370

[B27] Jashni M. K. Dols I. H. M. Iida Y. Boeren S. Beenen H. G. Mehrabi R. (2015). Synergistic action of a metalloprotease and a serine protease from *Fusarium oxysporum* f. sp. *lycopersici* cleaves chitin-binding tomato chitinases, reduces their antifungal activity, and enhances fungal virulence. *Mol. Plant Microbe Interact.* 28 996–1008. 10.1094/MPMI-04-15-0074-R 25915453

[B28] Jiménez-Ruiz J. Leyva-Pérez M. O. Cabanás C. G. L. Barroso J. B. Luque F. Mercado-Blanco J. (2019). The transcriptome of *Verticillium dahliae* responds differentially depending on the disease susceptibility level of the olive (*Olea europaea* L.) cultivar. *Genes (Basel)* 10:251. 10.3390/genes10040251 30934761 PMC6523120

[B29] Jin L. Chen D. Liao S. Zhang Y. Yu F. Wan P. (2019). Transcriptome analysis reveals downregulation of virulence-associated genes expression in a low virulence *Verticillium dahliae* strain. *Arch. Microbiol.* 201 927–941. 10.1007/s00203-019-01663-7 31020345

[B30] Jones P. Binns D. Chang H. Y. Fraser M. Li W. McAnulla C. (2014). InterProScan 5: genome-scale protein function classification. *Bioinformatics* 30 1236–1240. 10.1093/bioinformatics/btu031 24451626 PMC3998142

[B31] Jyothishwaran G. Kotresha D. Selvaraj T. Srideshikan S. H. Rajvanshi P. K. Jayabaskaran C. (2007). A modified freeze–thaw method for efficient transformation of *Agrobacterium tumefaciens*. *Curr. Sci.* 93 770–772.

[B32] Kaever A. Landesfeind M. Possienke M. Feussner K. Feussner I. Meinicke P. (2012). MarVis-filter: ranking, filtering, adduct and isotope correction of mass spectrometry data. *J. Biomed. Biotechnol.* 2012:263910. 10.1155/2012/263910 22550397 PMC3328170

[B33] Kersey P. J. Allen J. E. Allot A. Barba M. Boddu S. Bolt B. J. (2018). Ensembl genomes 2018: an integrated omics infrastructure for non-vertebrate species. *Nucleic Acids Res.* 46 D802–D808. 10.1093/nar/gkx1011 29092050 PMC5753204

[B34] Klosterman S. J. Atallah Z. K. Vallad G. E. Subbarao K. V. (2009). Diversity, pathogenicity, and management of *Verticillium* species. *Annu. Rev. Phytopathol.* 47 39–62. 10.1146/annurev-phyto-080508-081748 19385730

[B35] Klosterman S. J. Subbarao K. V. Kang S. Veronese P. Gold S. E. Thomma B. P. H. J. (2011). Comparative genomics yields insights into niche adaptation of plant vascular wilt pathogens. *PLoS Pathog.* 7:e1002137. 10.1371/journal.ppat.1002137 21829347 PMC3145793

[B36] Kolar M. Punt P. J. van den Hondel C. A. M. J. J. Schwab H. (1988). Transformation of *Penicillium chrysogenum* using dominant selection markers and expression of an *Escherichia coli lacZ* fusion gene. *Gene* 62 127–134. 10.1016/0378-1119(88)90586-0 3131191

[B37] Lenarčič T. Pirc K. Hodnik V. Albert I. Borišek J. Magistrato A. (2019). Molecular basis for functional diversity among microbial Nep1-like proteins. *PLoS Pathog.* 15:e1007951. 10.1371/journal.ppat.1007951 31479498 PMC6743777

[B38] Leonard M. Kühn A. Harting R. Maurus I. Nagel A. Starke J. (2020). *V. longisporum* elicits media-dependent secretome responses with a further capacity to distinguish between plant-related environments. *bioRxiv* [Preprint]. 10.1101/2020.02.11.943803 PMC7423881 32849460

[B39] Lievens B. Houterman P. M. Rep M. (2009). Effector gene screening allows unambiguous identification of *Fusarium oxysporum* f. sp. *lycopersici* races and discrimination from other *formae speciales*. *FEMS Microbiol. Lett.* 300 201–215. 10.1111/j.1574-6968.2009.01783.x 19799634

[B40] Lo Presti L. Lanver D. Schweizer G. Tanaka S. Liang L. Tollot M. (2015). Fungal effectors and plant susceptibility. *Annu. Rev. Plant Biol.* 66 513–545. 10.1146/annurev-arplant-043014-114623 25923844

[B41] Lombard V. Golaconda Ramulu H. Drula E. Coutinho P. M. Henrissat B. (2014). The carbohydrate-active enzymes database (CAZy) in 2013. *Nucleic Acids Res.* 42 D490–D495. 10.1093/nar/gkt1178 24270786 PMC3965031

[B42] Mandelc S. Javornik B. (2015). The secretome of vascular wilt pathogen *Verticillium albo-atrum* in simulated xylem fluid. *Proteomics* 15 787–797. 10.1002/pmic.201400181 25407791

[B43] Marton K. Flajsman M. Radisek S. Kosmelj K. Jakse J. Javornik B. (2018). Comprehensive analysis of *Verticillium nonalfalfae in silico* secretome uncovers putative effector proteins expressed during hop invasion. *PLoS One* 13:e0198971. 10.1371/journal.pone.0198971 29894496 PMC5997321

[B44] McCotter S. W. Horianopoulos L. C. Kronstad J. W. (2016). Regulation of the fungal secretome. *Curr. Genet.* 62 533–545. 10.1007/s00294-016-0578-2 26879194

[B45] Meinicke P. Lingner T. Kaever A. Feussner K. Göbel C. Feussner I. (2008). Metabolite-based clustering and visualization of mass spectrometry data using one-dimensional self-organizing maps. *Algorithms Mol. Biol.* 3:9. 10.1186/1748-7188-3-9 18582365 PMC2464586

[B46] Mösch H. U. Scheier B. Lahti R. Mäntsälä P. Braus G. H. (1991). Transcriptional activation of yeast nucleotide biosynthetic gene *ADE4* by GCN4. *J. Biol. Chem.* 266 20453–20456. 1939099

[B47] Mullins E. D. Chen X. Romaine P. Raina R. Geiser D. M. Kang S. (2001). *Agrobacterium*-mediated transformation of *Fusarium oxysporum*: an efficient tool for insertional mutagenesis and gene transfer. *Phytopathology* 91 173–180. 10.1094/phyto.2001.91.2.173 18944391

[B48] Naumann T. A. Price N. P. J. (2012). Truncation of class IV chitinases from *Arabidopsis* by secreted fungal proteases. *Mol. Plant Pathol.* 13 1135–1139. 10.1111/j.1364-3703.2012.00805.x 22512872 PMC6638631

[B49] Naumann T. A. Wicklow D. T. Price N. P. J. (2011). Identification of a chitinase-modifying protein from *Fusarium verticillioides*: truncation of a host resistance protein by a fungalysin metalloprotease. *J. Biol. Chem.* 286 35358–35366. 10.1074/jbc.M111.279646 21878653 PMC3195611

[B50] Neumann M. J. Dobinson K. F. (2003). Sequence tag analysis of gene expression during pathogenic growth and microsclerotia development in the vascular wilt pathogen *Verticillium dahliae*. *Fungal Genet. Biol.* 38 54–62. 10.1016/S1087-1845(02)00507-8 12553936

[B51] Oeser B. Heidrich P. M. Müller U. Tudzynski P. Tenberge K. B. (2002). Polygalacturonase is a pathogenicity factor in the *Claviceps purpurea*/rye interaction. *Fungal Genet. Biol.* 36 176–186. 10.1016/S1087-1845(02)00020-8 12135573

[B52] Perez-Riverol Y. Csordas A. Bai J. Bernal-Llinares M. Hewapathirana S. Kundu D. J. (2019). The PRIDE database and related tools and resources in 2019: improving support for quantification data. *Nucleic Acids Res.* 47 D442–D450. 10.1093/nar/gky1106 30395289 PMC6323896

[B53] Santhanam P. van Esse H. P. Albert I. Faino L. Nürnberger T. Thomma B. P. H. J. (2013). Evidence for functional diversification within a fungal NEP1-like protein family. *Mol. Plant Microbe Interact.* 26 278–286. 10.1094/MPMI-09-12-0222-R 23051172

[B54] Schmidt S. M. Houterman P. M. Schreiver I. Ma L. Amyotte S. Chellappan B. (2013). MITEs in the promoters of effector genes allow prediction of novel virulence genes in *Fusarium oxysporum*. *BMC Genomics* 14:119. 10.1186/1471-2164-14-119 23432788 PMC3599309

[B55] Scholz S. S. Schmidt-Heck W. Guthke R. Furch A. C. U. Reichelt M. Gershenzon J. (2018). *Verticillium dahliae-Arabidopsis* interaction causes changes in gene expression profiles and jasmonate levels on different time scales. *Front. Microbiol.* 9:217. 10.3389/fmicb.2018.00217 29497409 PMC5819561

[B56] Seidl M. F. Cook D. E. Thomma B. P. H. J. (2016). Chromatin biology impacts adaptive evolution of filamentous plant pathogens. *PLoS Pathog.* 12:e1005920. 10.1371/journal.ppat.1005920 27812218 PMC5094656

[B57] Sexton A. C. Minic Z. Cozijnsen A. J. Pedras M. S. C. Howlett B. J. (2009). Cloning, purification and characterisation of brassinin glucosyltransferase, a phytoalexin-detoxifying enzyme from the plant pathogen *Sclerotinia sclerotiorum*. *Fungal Genet. Biol.* 46 201–209. 10.1016/j.fgb.2008.10.014 19041410

[B58] Shevchenko A. Wilm M. Vorm O. Mann M. (1996). Mass spectrometric sequencing of proteins from silver-stained polyacrylamide gels. *Anal. Chem.* 68 850–858. 10.1021/ac950914h 8779443

[B59] Singh S. Braus-Stromeyer S. A. Timpner C. Tran V. T. Lohaus G. Reusche M. (2010). Silencing of *Vlaro2* for chorismate synthase revealed that the phytopathogen *Verticillium longisporum* induces the cross-pathway control in the xylem. *Appl. Microbiol. Biotechnol.* 85 1961–1976. 10.1007/s00253-009-2269-0 19826808 PMC2811248

[B60] Singh S. Braus-Stromeyer S. A. Timpner C. Valerius O. von Tiedemann A. Karlovsky P. (2012). The plant host *Brassica napus* induces in the pathogen *Verticillium longisporum* the expression of functional catalase peroxidase which is required for the late phase of disease. *Mol. Plant Microbe Interact.* 25 569–581. 10.1094/MPMI-08-11-0217 22112218

[B61] Smith G. (1949). The effect of adding trace elements to Czapek-Dox medium. *Trans. Br. Mycol. Soc.* 32 280–283. 10.1016/s0007-1536(49)80018-0

[B62] Sorrell T. C. Chen S. C. A. (2009). “Fungal-derived immune modulating molecules,” in *Pathogen-Derived Immunomodulatory Molecules. Advances in Experimental Medicine and Biology*, ed. Fallon P. G. (New York, NY: Springer), 108–120. 10.1007/978-1-4419-1601-3_9 20054979

[B63] Soyer J. L. El Ghalid M. Glaser N. Ollivier B. Linglin J. Grandaubert J. (2014). Epigenetic control of effector gene expression in the plant pathogenic fungus *Leptosphaeria maculans*. *PLoS Genet.* 10:e1004227. 10.1371/journal.pgen.1004227 24603691 PMC3945186

[B64] Subramanian S. Cho U. H. Keyes C. Yu O. (2009). Distinct changes in soybean xylem sap proteome in response to pathogenic and symbiotic microbe interactions. *BMC Plant Biol.* 9:119. 10.1186/1471-2229-9-119 19772575 PMC2758885

[B65] Tan K. C. Oliver R. P. (2017). Regulation of proteinaceous effector expression in phytopathogenic fungi. *PLoS Pathog.* 13:e1006241. 10.1371/journal.ppat.1006241 28426760 PMC5398718

[B66] Timpner C. Braus-Stromeyer S. A. Tran V. T. Braus G. H. (2013). The Cpc1 regulator of the cross-pathway control of amino acid biosynthesis is required for pathogenicity of the vascular pathogen *Verticillium longisporum*. *Mol. Plant Microbe Interact.* 26 1312–1324. 10.1094/MPMI-06-13-0181-R 23883358

[B67] Tran V.-T. Braus-Stromeyer S. A. Kusch H. Reusche M. Kaever A. Kühn A. (2014). *Verticillium* transcription activator of adhesion Vta2 suppresses microsclerotia formation and is required for systemic infection of plant roots. *New Phytol.* 202 565–581. 10.1111/nph.12671 24433459

[B68] van Esse H. P. Bolton M. D. Stergiopoulos I. de Wit P. J. G. M. Thomma B. P. H. J. (2007). The chitin-binding *Cladosporium fulvum* effector protein Avr4 is a virulence factor. *Mol. Plant Microbe Interact.* 20 1092–1101. 10.1094/mpmi-20-9-1092 17849712

[B69] Voigt C. A. Schäfer W. Salomon S. (2005). A secreted lipase of *Fusarium graminearum* is a virulence factor required for infection of cereals. *Plant J.* 42 364–375. 10.1111/j.1365-313X.2005.02377.x 15842622

[B70] Volk H. Marton K. Flajšman M. Radišek S. Tian H. Hein I. (2019). Chitin-binding protein of *Verticillium nonalfalfae* disguises fungus from plant chitinases and suppresses chitin-triggered host immunity. *Mol. Plant Microbe Interact.* 32 1378–1390. 10.1094/MPMI-03-19-0079-R 31063047

[B71] Wang J. Tian L. Zhang D. D. Short D. P. G. Zhou L. Song S. S. (2018). SNARE-encoding genes *VdSec22* and *VdSso1* mediate protein secretion required for full virulence in *Verticillium dahliae*. *Mol. Plant Microbe Interact.* 31 651–664. 10.1094/MPMI-12-17-0289-R 29419372

[B72] Wang J. Y. Cai Y. Gou J. Y. Mao Y. B. Xu Y. H. Jiang W. H. (2004). VdNEP, an elicitor from *Verticillium dahliae*, induces cotton plant wilting. *Appl. Environ. Microbiol.* 70 4989–4995. 10.1128/AEM.70.8.4989-4995.2004 15294839 PMC492334

[B73] Yadeta K. A. Thomma B. P. H. J. (2013). The xylem as battleground for plant hosts and vascular wilt pathogens. *Front. Plant Sci.* 4:97. 10.3389/fpls.2013.00097 23630534 PMC3632776

[B74] Yakoby N. Beno-Moualem D. Keen N. T. Dinoor A. Pines O. Prusky D. (2001). *Colletotrichum gloeosporioides* pelB is an important virulence factor in avocado fruit-fungus interaction. *Mol. Plant Microbe Interact.* 14 988–995. 10.1094/MPMI.2001.14.8.988 11497471

[B75] Yang Y. Zhang Y. Li B. Yang X. Dong Y. Qiu D. (2018). A *Verticillium dahliae* pectate lyase induces plant immune responses and contributes to virulence. *Front. Plant Sci.* 9:1271. 10.3389/fpls.2018.01271 30271415 PMC6146025

[B76] Yellareddygari S. K. R. Gudmestad N. C. (2018). Effect of soil temperature, injection depth, and rate of metam sodium efficacy in fine-textured soils with high organic matter on the management of *Verticillium wilt* of potato. *Am. J. Potato Res.* 95 413–422. 10.1007/s12230-018-9641-5

[B77] Zhang H. Yohe T. Huang L. Entwistle S. Wu P. Yang Z. (2018). dbCAN2: a meta server for automated carbohydrate-active enzyme annotation. *Nucleic Acids Res.* 46 W95–W101. 10.1093/nar/gky418 29771380 PMC6031026

[B78] Zhang Y. Gao Y. Liang Y. Dong Y. Yang X. Yuan J. (2017). The *Verticillium dahliae* SnodProt1-like protein VdCP1 contributes to virulence and triggers the plant immune system. *Front. Plant Sci.* 8:1880. 10.3389/fpls.2017.01880 29163605 PMC5671667

[B79] Zhou B. J. Jia P. S. Gao F. Guo H. S. (2012). Molecular characterization and functional analysis of a necrosis-and ethylene-inducing, protein-encoding gene family from *Verticillium dahliae*. *Mol. Plant Microbe Interact.* 25 964–975. 10.1094/MPMI-12-11-0319 22414440

