# Supplementary figures and images for: Verticillium longisporum Elicits Media-Dependent Secretome Responses With Capacity to Distinguish Between Plant-Related Environments
Source: Front Microbiol. 2020 Aug 6;11:1876. doi: 10.3389/fmicb.2020.01876 (PMC7423881; doi:10.3389/fmicb.2020.01876)

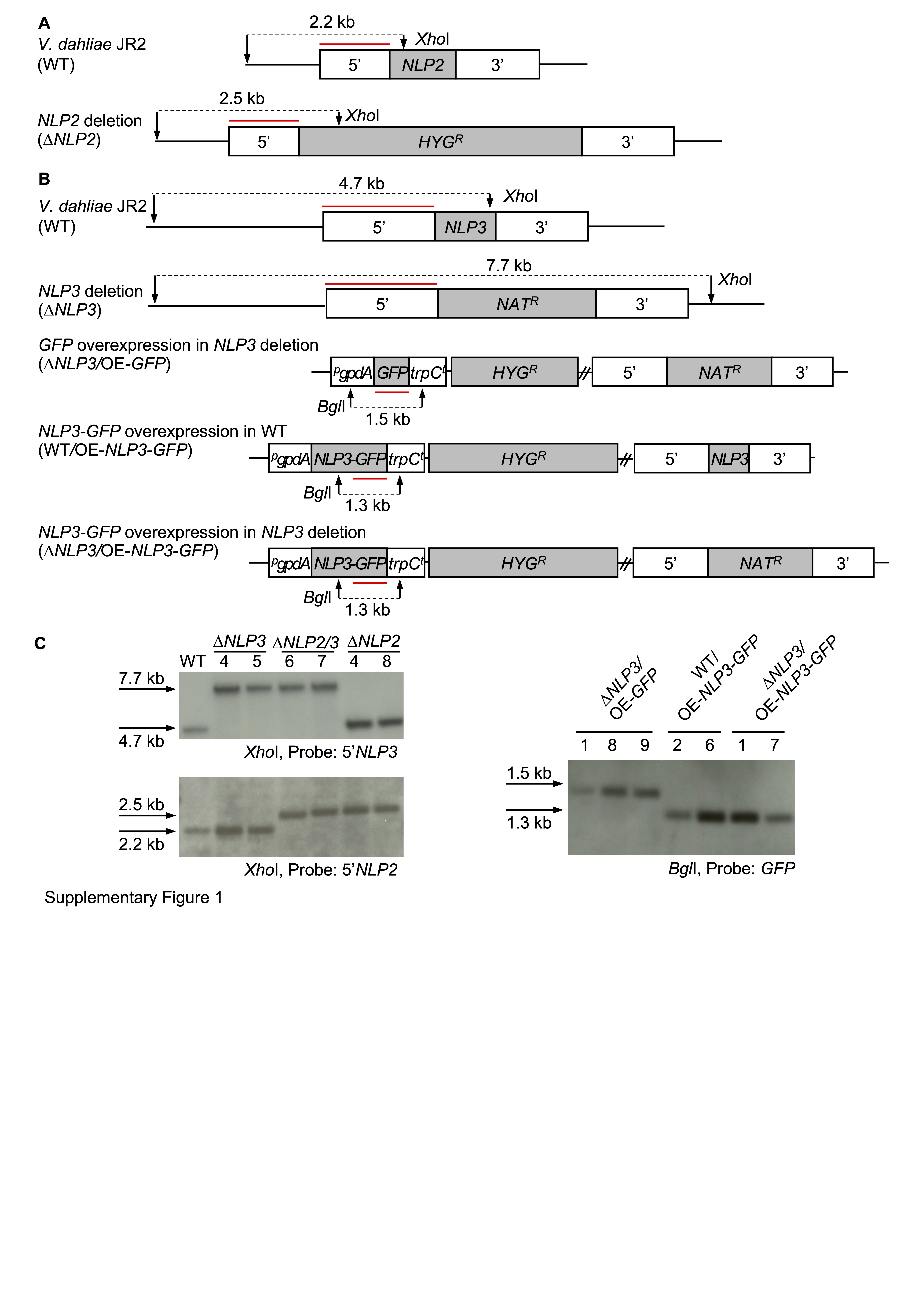

Supplement: FIGURE S1 — Verification of V. dahliae NLP2 and NLP3 deletion or overexpression constructs. [file Image_1.JPEG]

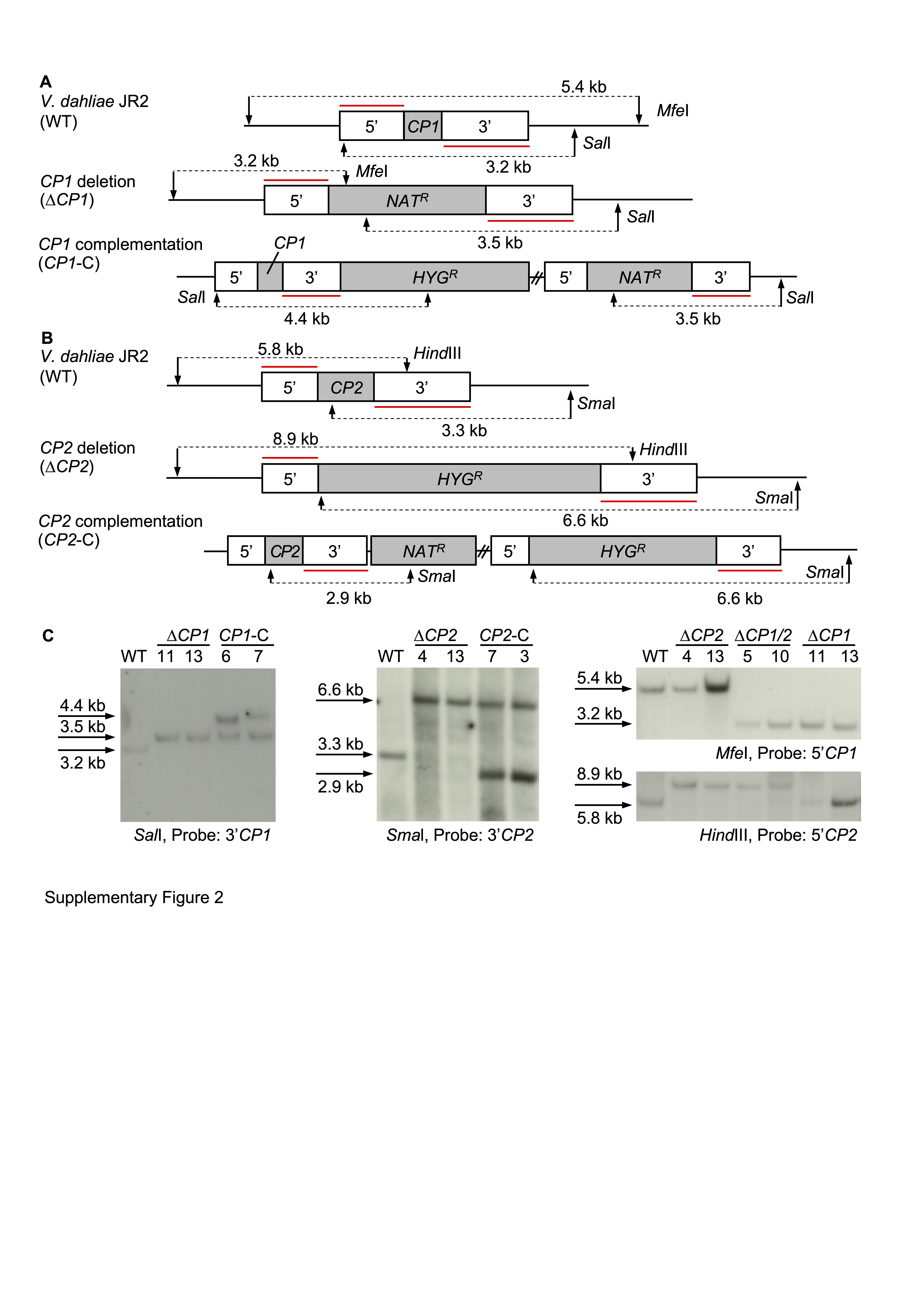

Supplement: FIGURE S2 — Verification of V. dahliae CP1 and CP2 deletion and complementation strains. [file Image_2.JPEG]

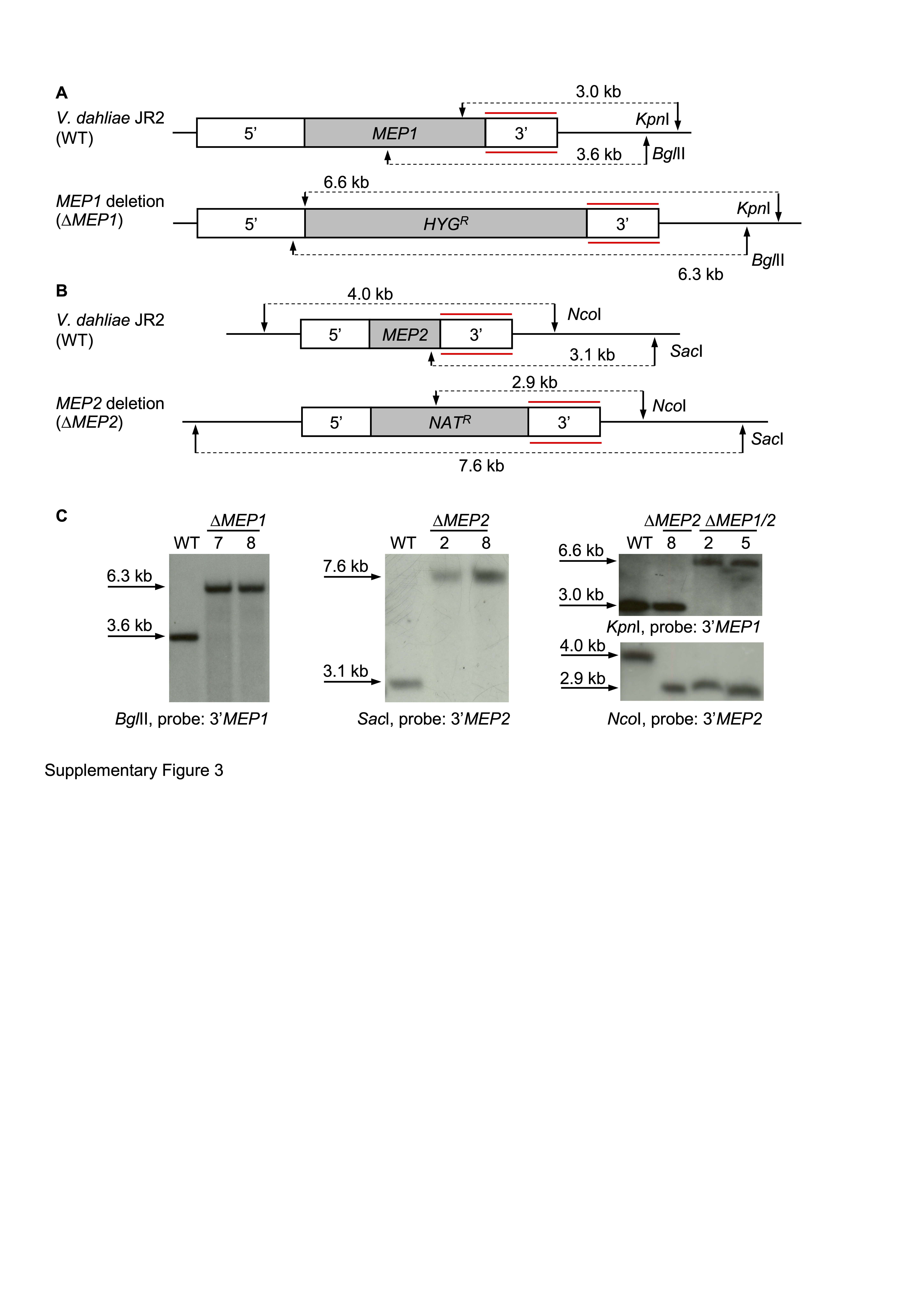

Supplement: FIGURE S3 — Verification of V. dahliae MEP1 and MEP2 deletion strains. [file Image_3.JPEG]

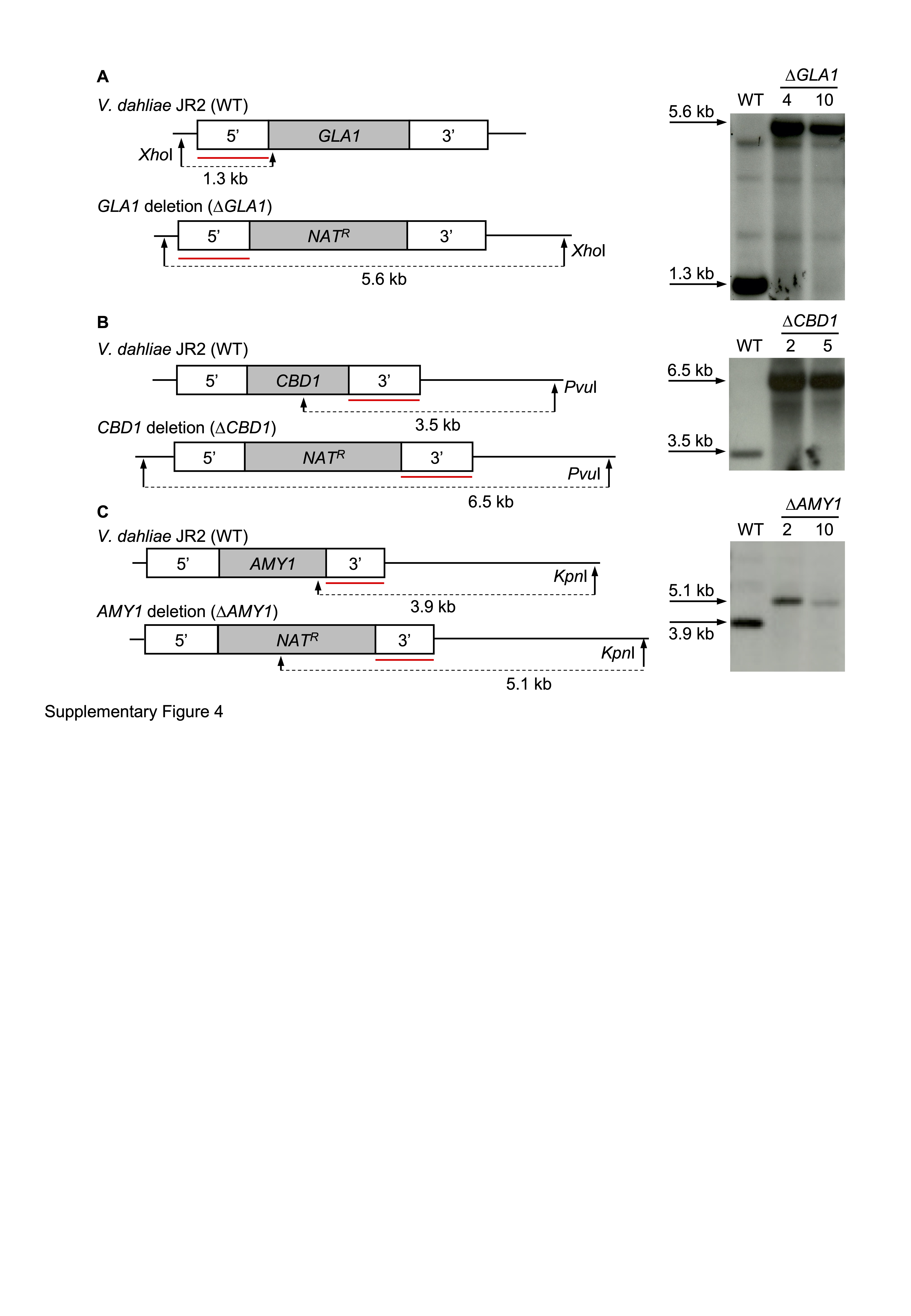

Supplement: FIGURE S4 — Verification of V. dahliae GLA1, CBD1, and AMY1 deletion strains. [file Image_4.JPEG]

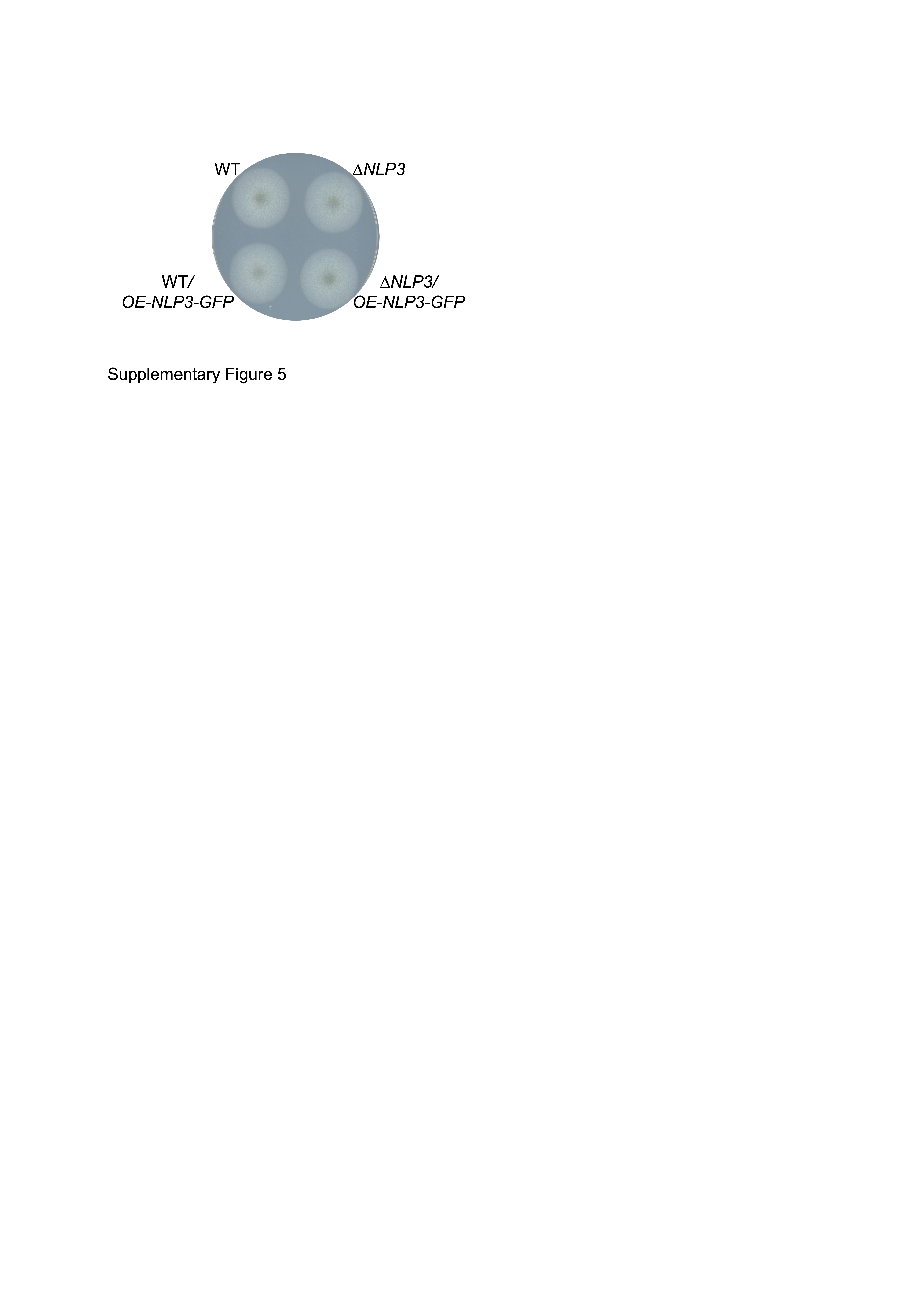

Supplement: FIGURE S5 — Expression of NLP3-GFP under control of a constitutively active promoter allows V. dahliae wildtype-like growth on solid media. [file Image_5.JPEG]
