# Supplementary material for: Verticillium longisporum Elicits Media-Dependent Secretome Responses With Capacity to Distinguish Between Plant-Related Environments
Source: Front Microbiol. 2020 Aug 6;11:1876. doi: 10.3389/fmicb.2020.01876 (PMC7423881; doi:10.3389/fmicb.2020.01876)
Supplement: TEXT S1 — Additional materials and methods. [file Data_Sheet_1.docx]

Supplementary Material

# Supplementary Data

## Supplementary Text

**Supplementary Text 1. Additional Materials and Methods.**

The following plasmids were constructed for the use of the backbone or resistance marker cassettes. The plasmid pPK2 was cut with *Sac*I and *Xba*I to receive the backbone. The hygromycin resistance gene and *trpC* promoter were amplified from pSilent-1 with primer pair HygCAS-F/HygCAS-R. This fragment was cut with *Sac*I and *Xba*I and ligated to the backbone resulting in pME4564 (pKO1). This plasmid was solely used as backbone donor. For pKO2, pME4564 was cut with *Sac*I and *Apa*I to dismiss the hygromycin resistance cassette and the backbone was ligated to the *gpdA* promoter and nourseothricin resistance gene, which was excised from pNAT1 by cutting with *Sac*I and *Apa*I. To attach a terminator to the resistance gene, the *trpC* terminator was amplified from pPK2 using primers ML7/ML8 and the *gpdA* promoter and the nourseothricin resistance marker gene from pKO2 were amplified with the primers ML5/ML6. The fragments were fused by PCR with primers ML8/ML9 and cloned into the pJet1.2 Cloning vector, resulting in pME4978. To construct pME4815, the backbone of pME4564 was amplified using the primers ML1/ML2. The nourseothricin marker cassette was amplified from pME4978 with the primers SAB52/SAB53 and fused to the pME4564 backbone via Seamless cloning, resulting in pME4815.

**Gene deletion and overexpression in the haploid *V. dahliae***

For deletion of *NLP3* (*VDAG_JR2_Chr4g05950a*) ORF the 5’ and 3’ flanking regions were amplified from *V. dahliae* WT gDNA with ML86/ML87 (1 561 bp) and ML88/ML89 (1 284 bp), respectively. The nourseothricin resistance cassette was obtained by amplification of pME4815 with ML8/ML9 (2 194 bp). pME4564 was cut with *Stu*I and *Eco*RV and the linearized backbone (6 804 bp) was ligated with the fragments (*5’NLP3*, nourseothricin resistance cassette and 3’*NLP3*) resulting in pME4883. *V. dahliae* JR2 WT was transformed with this construct resulting in *NLP3* deletion strains (VGB384, VGB385). Transformants were tested for correct integration of the construct by Southern hybridization (Supplementary Figure 1). The gDNA of WT and transformant strains was treated with *Xho*I and the 5’ flanking region of *NLP3* was prepared as probe to verify the *NLP3* deletion strains.

Additionally, strains expressing free *GFP* or *NLP3-GFP* were constructed. The *NLP3* deletion strain VGB384 was transformed with pGreen2 (Tran *et* al., 2014) to generate the *NLP3* deletion strain expressing free *GFP*. Transformants were screened for their fluorescence signal by confocal microscopy after growing fungal hyphae in µ-slide 8 well microcopy chambers (Ibidi) at 25°C overnight. Selected transformants were next tested by Southern hybridization to possess the ectopically integrated fragments (Supplementary Figure 1). Genomic DNA of WT and the transformants was cut by *Bgl*I. Correct strains were verified with *GFP* as a probe. VGB431 and VGB432 were conserved. To overexpress C-terminally tagged *NLP3* in the WT or *NLP3* deletion strain, an *NLP3-GFP* possessing construct was generated including the hygromycin resistance cassette and integrated into WT or the *NLP3* deletion strain VGB384, respectively. The *gpdA* promoter was amplified from pGreen2 with ML99/RH635 (874 bp), the *NLP3* sequence without the stop codon was amplified from genomic WT DNA with ML100/ML101 (874 bp). ML101 includes a sequence coding for GGSGG that serves as a flexible linker to allow independent protein folding (van Rosmalen et al., 2017). These fragments were fused in another PCR using primer pair ML101/RH635. *GFP* (without the start codon) fused to *trpC* terminator was obtained by PCR of pGreen2 with RH686/RH636 (1 507 bp). These fragments were ligated to *Eco*RV linearized pPK2. The constructed plasmid was named pME4990. This plasmid was used to transform the desired fragment into *V. dahliae* WT and VGB384. Transformants were tested by Southern hybridization as VGB431 an VGB432 (Supplementary Figure 1). VGB407 and VGB408 were verified to possess the *NLP3-GFP* in the WT and VGB409 and VGB410 showed positive signals for the construct in the *NLP3* deletion strain.

To generate an *NLP2* (*VDAG_JR2_Chr2g05460a*) deletion strain, the 5’ and 3’ flanking regions were amplified with ML90/ML91 (975 bp) and ML92/ML106 (1 165 bp), respectively, from WT gDNA and the *HYG^R^* cassette with ML8/RO3 (3 942 bp) of pPK2 (Covert et al., 2001). Plasmid pME4564 was cut with *Stu*I and *Eco*RV and the linearized backbone (6 804 bp) was ligated with the fragments resulting in pME4885. *V. dahliae* WT and *NLP3* deletion strain were transformed with the construct and by homologous recombination the *NLP2* deletion strain and *NLP2/NLP3* double deletions strains were generated, respectively. Transformants were verified by Southern hybridization (Supplementary Figure 1). Verification of *NLP2* single and *NLP2/3* double deletion strains was obtained by processing gDNA with *Xho*I and 5’ *NLP3* and 5’ *NLP2* were used as probes to confirm VGB390, VGB391, VGB400 and VGB401.

For construction of the *CP1* (*VDAG_JR2_Chr7g00860a*) deletion strain, the 5’ and 3’ flanking regions were amplified of WT gDNA with AO84/AO85 (891 bp) and AO86/AO87 (1 205 bp), respectively. The nourseothricin resistance cassette was fused together with 5’ and 3’flanking regions in a fusion PCR with AO84/AO87 (4 291 bp), which was then phosphorylated with T4 Polynucleotide Kinase (Thermo Fisher Scientific). The backbone of pME4564 (6 804 bp) was received by enzyme restriction with *Eco*RV and *Stu*I. It was dephosphorylated with FastAP Thermosensitive Alkaline Phosphatase (Thermo Fisher Scientific) and ligated to the insert with T4 DNA Ligase. *V. dahliae* JR2 was transformed with the resulting plasmid pME4887 to generate the *CP1* deletion strain. For the construct of the complementation strain the *CP1* ORF was amplified including the 5’ and 3’ flanking regions with AO131/AO132 (2 626 bp) and ligated to *Eco*RV linearized pPK2 (10 751 bp) resulting in pME4888. The complementation was ectopically integrated into *CP1* deletion strain. To verify the constructed strains they were subjected to Southern hybridization (Supplementary Figure 2). For confirmation of *CP1* deletion strains VGB316 and VGB317 and *CP1* complementation strains VGB489 and VGB490 gDNA of WT and the transformants was cut with *Sal*I while 3’ *CP1* region was used as probe.

Construction of *CP2* (*VDAG_JR2_Chr2g07000a*) deletion strain required amplification of 5’ and 3’ flanking region of *CP2* with ML94/ML95 (778 bp) and ML96/ML97 (1 343 bp), respectively, of WT gDNA. Together with the *HYG^R^* cassette the fragments were ligated to the backbone of pME4564, which was linearized with *Stu*I and *Eco*RV (6 804 bp). The resulting plasmid pME4889 was used to transform *V. dahliae* WT resulting in the *CP2* deletion strains VGB406 and VGB422. For the complementation the plasmid pME4890 was constructed by amplifying the *CP2* ORF including its 5’ and 3’ flanking regions with ML104/ML105 (2 913 bp) of WT gDNA, which was ligated to *Eco*RV linearized pME4815. This construct was utilized to transform *CP2* deletion strain VGB406 resulting in the ectopic complementation strains VGB429 and VGB430. Additionally, VGB406 was transformed with pME4887 to generate the ∆*CP1*∆*CP2* double deletion strains VGB423 and VGB424. All strains were confirmed in Southern hybridizations (Supplementary Figure 2). To test *CP2* strains, gDNA was cut with *Sma*I and correct fragments were detected with 3’ *CP2* region as probe resulting in the verification of *CP2* deletion and ectopic complementation strains. For verification of ∆*CP1*∆*CP2* strains gDNA was cut with *Mfe*I or *Hind*III and as probes the 5’ flanking region of *CP1* or *CP2* were utilized, respectively.

For the deletion of *MEP1* (*VDAG_JR2_Chr8g09760a*) the annotation of VdLs.17 *MEP1* (*VDAG_03418*) starting 341 bp upstream of the JR2 prediction was considered in addition to the prediction for *V. dahliae* JR2 to ensure the whole gene is deleted. The corresponding 5’ and 3’ flanking regions were amplified of WT gDNA with JST27/JST28 (1 500 bp) and JST29/JST30 (1 000 bp), respectively. The fragments were ligated together with the *HYG^R^* cassette to the pME4564 backbone, which was obtained by PCR amplification with ML1/ML2 (6 727 bp), resulting in pME4891. *V. dahliae* WT was transformed with the deletion construct and *MEP1* deletion strains VGB226 and VGB227 were confirmed by Southern hybridization (Supplementary Figure 3). Genomic DNA was cut with *Bgl*II and *MEP1* 3’ flanking region was used as probe.

To generate the *MEP2* (*VDAG_JR2_Chr1g21900a*) deletion strain the 5’ and 3’ flanking regions were amplified with ML12/ML13 (960 bp) and ML14/ML15 (1 000 bp) of WT gDNA, respectively. Together with the *NAT^R^* cassette the fragments were ligated to the backbone of pME4564, which was generated by PCR amplification with ML1/ML2 (6 727 bp). The resulting plasmid pME4892 was used for construction of *MEP2* deletion strain by transformation of *V. dahliae* WT. Genomic DNA of transformants and WT was processed with *Sac*I and *MEP2* deletion strains VGB126 and VGB133 were confirmed by Southern hybridization with 3’ *MEP2* as probes (Supplementary Figure 3). Strain VGB126 was further transformed with pME4891 to obtain the double deletion strains *∆MEP1∆MEP2* VGB203 and VGB204 that were verified by Southern hybridization with *Kpn*I treated gDNA and 3’ *MEP1* or *Nco*I treated gDNA and 3’ *MEP2*.

To obtain plasmids for the generation of deletion strains with a *NAT^R^* cassette, the 5’ and 3’ flanking regions of the respective genes were amplified of *V. dahliae* WT gDNA. Fragments were then ligated to the backbone of pME4564 that was gained by PCR amplification with ML1/ML2 (6 727 bp).

The *GLA1* (*VDAG_JR2_Chr8g11020a*) deletion construct pME4894 contains the 5’ and 3’ flanking regions amplified with ML20/ML21 (990 bp) and ML22/ML23 (1 095 bp), respectively. After transformation of *V. dahliae* WT with this construct, the resulting transformants were tested for their correct insert by Southern hybridization with *Xho*I treatment of the gDNA and 5’ flanking region as probe (Supplementary Figure 4). The confirmed *GLA1* deletion strains VGB129 and VGB130 were conserved.

For the *CBD1* (*VDAG_JR2_Chr4g04440a*) deletion construct the 5’ and 3’ flanking regions were amplified with ML16/ML17 (982 bp) and ML18/ML19 (996 bp), respectively. The resulting plasmid pME4895 was used to generate *CBD1* deletion strains. VGB127 and VGB132 were confirmed by Southern hybridization (Supplementary Figure 4). The gDNA was cut with *Pvu*I and 3’ flanking region of *CBD1* was used as probe.

Construction of *AMY1* (*VDAG_JR2_Chr7g03330a*) deletion construct involves amplification of the 5’ and 3’ flanking regions with ML24/ML25 (1 013 bp) and ML26/ML27 (814 bp), respectively. The construct pME4896 was utilized for transformation of *V. dahliae* WT resulting in *AMY1* deletion strains VGB128 and VGB130. The strains were verified by Southern hybridization with *Kpn*I treated gDNA and 3’ flanking region as probe (Supplementary Figure 4).

## Supplementary Figures

**Supplementary Figure 1. Verification of *V. dahliae NLP2* and *NLP3* deletion or overexpression constructs.** *V. dahliae* JR2 (WT) was transformed with the respective deletion cassettes to generate *NLP2* and *NLP3* deletion strains (∆*NLP2* and ∆*NLP3*, respectively) via homologous recombination. The *NLP2/NLP3* double deletion strain (∆*NLP2/3*) was obtained by transforming the ∆*NLP3* strain with the ∆*NLP2* construct*.* Strains overexpressing ectopically integrated *GFP* or *NLP3-GFP* were constructed by transforming WT or *NLP3* deletion strains with the displayed constructs (*GFP* and *NLP3-GFP* are driven by the *gpdA* promoter and followed by the *trpC* terminator). All constructs contain resistance cassettes (*HYG^R^*: hygromycin resistance; *NAT^R^*: nourseothricin resistance) with a *gpdA* promoter and a *trpC* terminator. Schemes with used restriction enzymes, corresponding cutting sites (arrows) and probes (red lines) used for Southern hybridization are presented in **(A)** and **(B)**. **(A)** *XhoI* and the 5’ flanking region as probe were used for verification of the *NLP2* deletion strain*.* **(B)** ∆*NLP3* strain was confirmed using *Xho*I with 5’ flanking region as probe. And ectopic integration of *GFP* into ∆*NLP3* strain and *NLP3-GFP* into WT or ∆*NLP3* strain was verified with restriction enzyme *Bgl*I with *GFP* as probe. **(C)** Confirmation of deletion strains by Southern hybridization is shown for ∆*NLP2* #4 = VGB390, #8 = VGB391; ∆*NLP3* #4 = VGB384, #5 = VGB385, ∆*NLP2/3* #6 = VGB400, #7 = VGB401; ∆*NLP3/*OE*-GFP* #8 = VGB431, #9 = VGB432, WT/OE-*NLP3-GFP* #2 = VGB407, #6 = VGB408, ∆*NLP3/*OE*-NLP3-GFP* #1 = VGB409, #7 = VGB410. Genomic WT DNA served as control. Restriction enzymes, probes and sizes of expected fragments are indicated.

**Supplementary Figure 2. Verification of *V. dahliae* *CP1* and *CP2* deletion and complementation strains.** Deletion strains (∆*CP1 and* ∆*CP2*) were obtained via homologous recombination between the deletion constructs and *V. dahliae* JR2 (WT). The *CP2* deletion strain was transformed with the *CP1* deletion construct to generate the double deletion strain ∆*CP1/2*. To generate ectopic complementation strains the constructs were integrated into the deletion strain as indicated by //. The constructs contain resistance cassettes (*HYG^R^*: hygromycin resistance; *NAT^R^*: nourseothricin resistance) controlled by the *gpdA* promoter and the *trpC* terminator. Schemes with used restriction enzymes, corresponding cutting sites (arrows) and probes (red lines) used for Southern hybridization are presented in **(A)** and **(B)**. **(A)** Confirmation of *CP1* deletion and complementation strain was achieved by enzyme restriction of gDNA with *Sal*I and 3’ flanking region as probe or *Mfe*I and 5’ flanking region as probe. **(B)** *Sma*I with 3’ flanking region or *Hind*III with 5’ flanking region as probe was used for *CP2* deletion and complementation strains. **(C)** Deletion and complementation strains were verified by Southern hybridization: ∆*CP1 #*11 = VGB316, #13 = VGB317; *CP1*-C #6 = VGB489, #7 = VGB490; ∆*CP2* #4 = VGB406, #14 = VGB422; *CP2*-C #3 = VGB429, #7 = VGB430; ∆*CP1*∆*CP2* #5 = VGB423, #10 = VGB424. Genomic WT DNA was used as control. Restriction enzymes, probes and sizes of expected fragments are depicted.

**Supplementary Figure 3. Verification of *V. dahliae MEP1* and *MEP2* deletion strains.** *MEP1* and *MEP2* deletion strains (∆*MEP1* and ∆*MEP2*, respectively) were constructed via homologous recombination between the deletion construct and *V. dahliae* JR2 (WT) and confirmed by Southern hybridization. Restriction sites of used enzymes (arrows) and probes (red lines) used for Southern hybridization are depicted in **(A)** and **(B)**. **(A)** Deletion of *MEP1* was confirmed after enzyme restriction of gDNA with *Kpn*I or *Bgl*II and 3’ flanking region as probe. *MEP1* deletion construct contains hygromycin resistance cassette (*HYG^R^*) controlled by the *gpdA* promoter and the *trpC* terminator. **(B)**Verification of *MEP2* deletion strain containing the nourseothricin resistance (*NAT^R^*) cassette under control of the *gpdA* promoter and the *trpC* terminator was achieved with *Sac*I or *Nco*I restriction and 3’ flanking region as probe. **(C)** Deletion strains were confirmed by Southern hybridization: ∆*MEP1* #7 = VGB226, #8 = VGB227; ∆*MEP2* #2 = VGB133, #8 = VGB126; ∆*MEP1/2* #2 = VGB204, #5 = VGB203. Genomic WT DNA served as control. Restriction enzymes, probes and sizes of expected fragments are indicated.

**Supplementary Figure 4. Verification of *V. dahliae* *GLA1*, *CBD1* and *AMY1* deletion strains.** By homologous recombination between the deletion construct and *V. dahliae* JR2 (WT) the depicted deletion strains were obtained. The deletion constructs contain a nourseothricin resistance cassette (*NAT^R^*) controlled by the *gpdA* promoter and the *trpC* terminator. The used probes (red lines) and restriction enzymes with their cutting sites (arrows) are illustrated in the schemes (left). The expected fragments were confirmed by Southern hybridization for each deletion strain with WT as control (right). **(A)** With *Xho*I and 5’ flanking region as probe *GLA1* deletion strains (*∆GLA1*) #4 = VGB129 and #10 = VGB131 were verified. **(B)** Verification of *CBD1* deletion strains (*∆CBD1* #2 = VGB127, #5 = VGB132) was achieved with *Pvu*I enzyme restriction and 3’ flanking region as probe and **(C)** *AMY1* deletion strains (*∆AMY1* #2 = VGB130, #10 = VGB128) were verified with *Kpn*I and 3’ flanking region as probe.

**Supplementary Figure 5. Expression of *NLP3-GFP* under control of a constitutively active promoter allows *V. dahliae* wildtype-like growth on solid media.** 50 000 spores of *V. dahliae* JR2 wildtype (WT) and *NLP3* deletion strains (*∆NLP3*) and corresponding strains ectopically overexpressing *NLP3-GFP* (WT/*OE-NLP3-GFP* and *∆NLP3*/*OE-NLP3-GFP*, respectively) were point inoculated on minimal medium (CDM) plates. The growth phenotype was observed after ten days of incubation at 25°C and revealed a similar phenotype for all tested strains.

## Supplementary Tables

**Supplementary Table 1. List of identified proteins in SXM and xylem sap.**

**Supplementary Table 2. List of other identified proteins without predicted secretion in xylem sap.**

**Supplementary Table 3. List of other identified proteins without predicted secretion in SXM.**

**Supplementary Table 4. Annotation of identified proteins in SXM and xylem sap.**

**Supplementary Table 5. Functional groups of secreted proteins.**

**Supplementary Table 6. CAZyme classification of secreted proteins.**

**Supplementary Table 7. *Verticillium* strains used in this study.**

**Supplementary Table 8. Primers used in this study.**

**Supplementary Table 9. Plasmids used in this study.**

**Supplementary Table 10. Mann-Whitney Test.**

**Supplementary Text 1. Additional Materials and Methods.**
